# Supplementary material for: Blue-shift photoconversion of near-infrared fluorescent proteins for labeling and tracking in living cells and organisms
Source: Nat Commun. 2023 Dec 19;14:8402. doi: 10.1038/s41467-023-44054-9 (PMC10730883; doi:10.1038/s41467-023-44054-9)
Supplement: Supplementary file 1 — Supplementary Information [file 41467_2023_44054_MOESM1_ESM.pdf]

Supplementary Information for “Blue-shift photoconversion of near-infrared fluorescent proteins for labeling and tracking in living cells and organisms”

Francesca Pennacchietti[a*], Jonatan Alvelid[a], Rodrigo A. Morales[b,c], Martina Damenti[a], Dirk Ollech[a], Olena S. Oliinyk[d], Daria M. Shcherbakova[e], Eduardo J. Villablanca[b,c], Vladislav V. Verkhusha[d,e], and Ilaria Testa[a,*]

[a] *Department of Applied Physics and SciLifeLab, KTH Royal Institute of Technology, Stockholm 17165, Sweden*

[b] *Division of Immunology and Allergy, Department of Medicine Solna, Karolinska Institutet and University Hospital, Stockholm 17176, Sweden*

[c] *Center for Molecular Medicine, Stockholm 17176, Sweden*

[d] *Medicum, University of Helsinki, Helsinki 00290, Finland*

[e] *Department of Genetics, and Gruss-Lipper Biophotonics Center, Albert Einstein College of Medicine, Bronx, New York 10461, USA*

* Correspondence to Ilaria Testa (testa@kth.se) and Francesca Pennacchietti (frapen@kth.se)

Supplementary table 1. Experimental settings for the presented data.

Figure	Channel	λ	I (kW/cm^2)	Δλ	dwell time (µs)	Pixel size (nm)	Line scan	System/structure
**1a**	Blue-ch	590	1.5 kW/cm2	600-640	3.16	72	1	*E. coli* – miRFP709
	Red-ch	670	3.18 kW/cm2	710-750			1	
	PC	700	0.8 MW/cm2<br>(~0.03 – 2.6 MW/cm2)	-			1	
**1d, S4a**	Blue-ch	590	1.2 kWcm2	600 – 640	3.16	72	1	*E. coli* – miRFP709
	Red-ch	670	1.6 kW/cm2	710-750			1	
	PC	670-800	~0.02 – 5 MW/cm^2	-			1	
**1e, S4b**	Blue-ch	550<br>570<br>570<br>590<br>590	0.12 kW/cm2<br>0.25 kW/cm2<br>0.25 kW/cm2<br>0.9 kW0/cm2<br>0.6 kW/cm2	560 – 600<br>580 – 620<br>580 – 620<br>610 – 650<br>600 – 640	3.16	72	1	*E. coli* – miRFP670, miRFP703, miRFP709, miRFP713, miRFP720
	Red-ch	640<br>670<br>670<br>670<br>670	1.08 kW/cm2<br>1.4 kW/cm2<br>1.4 kW/cm2<br>1.2 kW/cm2<br>1.1 kW/cm2	650 – 700<br>700 – 750<br>700 – 750<br>700 – 750<br>710 – 750			1	
	PC	670 – 800	0.93 MW/cm2	-			1	
**1f, S7**	Blue-ch	560<br>590<br>590<br>610<br>610	0.12 kW/cm2<br>0.25 kW/cm2<br>0.25 kW/cm2<br>0.9 kW0/cm2<br>0.6 kW/cm2	570 – 600<br>600 – 640<br>600 – 640<br>620 – 660<br>620 – 660	3.16	72	1	*E. coli* – miRFP670, miRFP703, miRFP709, miRFP713, miRFP720
	Red-ch	640<br>660<br>660<br>660<br>670	1.08 kW/cm2<br>1.4 kW/cm2<br>1.4 kW/cm2<br>1.2 kW/cm2<br>1.1 kW/cm2	650 – 690<br>710 – 750<br>710 – 750<br>710 – 750<br>710 – 750			1	
	PC	700<br>740<br>740<br>760<br>740	~ 2MW/cm^2	-			1	
**1g, S8**	Blue-ch	590	0.9 kW/cm^2	600-650	15.4	0.181 (xy)<br>1 (z)	1	*E. coli* – miRFP713
	Red-ch	670	1.1 kW/cm^2	700-795			1	
	PC	775 (120fs)<br>405<br>775 (700ps)	1.6 MW/cm^2<br>0.4 MW/cm^2<br>7.9 MW/cm^2	-	7.7	45 nm	1<br>1<br>4	
**S5**	Blue-ch	640	2.3 kW/cm2	670/40	50	58.6	1	*E. coli* – miRFP720
	Red-ch	640	2.3 kW/cm2	725/40			1	
	PC	775 (550 ps)	0.07 – 50 MW/cm2<br>Image at 21 MW/cm2	-			1	
**S6**	Blue-ch	590	1.5 kW/cm2	600-640	3.16	72	1	*E. coli* – miRFP709
	Red-ch	670	3.18 kW/cm2	710-750			1	
	PC	700	775 nm = 0.03 – 2.6 MW/cm2<br>405 nm = 0.03 – 2.5 MW/cm2	-			1	
**S13**	Blue-ch	640	50 kW/cm2	670/40	50	58.6	1	U2OS, vimentin-miRFP
	Red-ch	640	50 kW/cm2	725/40			1	
	PC	775 (550 ps)	0.07 – 25 MW/cm2	-			1	
**S9**	Blue-ch	640	2.3 kW/cm2	670/40	50	58.6	1	*E. coli* – miRFP720
	Red-ch	640	2.3 kW/cm2	725/40			1	
	PC	775 (550 ps)	21 MW/cm2	-			1	
**S10**	Blue-ch	640	2.3 kW/cm2	670/40	50	58.6	1	*E. coli* – miRFP720

	Red-ch	640	2.3 kW/cm2	725/40			1	
	PC	775 (550 ps)	0.07 – 50 MW/cm2 Panel (a) at 21 MW/cm2	-			1	
**S11**	Blue-ch	594	4.8 kW/cm2	620 – 650	3.16	57	1	Hela cells, H2B /Vimentin / LAMP1–miRFP720
	Red-ch	633 / 670	2.6 - 4.8 kW/cm2	710 – 750			1	
	PC	405	0.21 MW/cm2	-			16	
**2a, b**	Blue-ch	594	4.8 kW/cm2	620 – 650	3.16	57	1	Hela cells, H2B – miRFP720
	Red-ch	633	2.6 kW/cm2	710 – 750			1	
	PC	405	0.21 MW/cm2	-			16	
**2c – g**	Blue-ch/Red-ch	594	4.8 kW/cm2	620 – 740 (Δ = 20 nm)	3.16	114	1	Hela cells, H2B-miRFP720, E. coli miRFP720
	PC	405 nm	0.21 MW/cm2	-		57	16	
**3e**	Blue-ch	590	1.8 kW/cm2	600 – 650	15.4	114	1	*E. coli*, miRFP713
	Red-ch	670	4.8 kW/cm2	680 – 750			1	
	Blue-ch	488	0.06 kW/cm2	500 – 530			1	*E. coli*, Dendra2
	Red-ch	561	0.27 kW/cm2	570 – 600			1	
	PC	775 nm (700 ps) + 488 nm	7.9 MW/cm2 0.13 kW/cm2	-	30.8	57	64	
**3d**	Blue-ch	590	1.8 kW/cm2	600 – 650	15.4	114	1	*E. coli*, miRFP713
	Red-ch	670	4.8 kW/cm2	680 – 750			1	
	PC	775 (700 ps)	7.9 MW/cm2	-	30.8	57	6	
	Blue-ch	488	0.06 kW/cm2	500 – 530	15.4	114	1	*E. coli*, Dendra2
	Red-ch	561	0.27 kW/cm2	570 – 600			1	
	PC	405	0.1 kW/cm2	-	15.4	57	1	
**4e, S11, S12**	Blue-ch	610	5.2 kW/cm2	620 – 650	7.8	57	1	Hela cells, mEos3.2-Clathrin, Dendra2-CD9, LAMP1-miRFP720
	Red-ch	670	4.8 kW/cm2	710 – 750			1	
	Blue-ch	488	1.3 – 5 kW/cm2	500 – 550			1	
	Red-ch	561	0.5 – 1 kW/cm2	570 – 600			1	
	PC	405	0 – 0.2 MW/cm2	-			1-16	
**4b, 4g-i, S15, S16**	Blue-ch	594	4.8 kW/cm2	620 – 640	15.4	38.75	1	Hela cells, LAMP1 – miRFP720, mEos2-Peroxisomes
	Red-ch	633	7.2 kW/cm2	710 – 800			1	
	Blue-ch	488	1.3 kW/cm2	500 – 550			1	
	Red-ch	561	0.4 kW/cm2	596 – 600			1	
	PC	405	0.21 MW/cm2	-			1	
**5, S17, S18**	Blue-ch	640	6.2 kW/cm2	670/40	50	30 nm (sted) /120 nm (confocal)	1	U20S cells, vimentin-miRFP720 and H2B-miRFP720
	Red-ch			725/40			1	
	PC	775 (550 ps)	3.95 MW/cm2	-		30 nm	1	
**6, S19**	Blue-ch	640	6.2 kW/cm2	670/40	100	100 (xy), 400 (z)	1	Zebrafish, H2B-miRFP720
	Red-ch			725/40			1	
	PC	775 (550 ps)	3.95 MW/cm2	-		30 nm	1	
**S20**	Blue-ch	590	1.8 kW/cm2	600 - 650	102.4	303	1	Zebrafish, H2B-miRFP720
	Red-ch	670	4.8 kW/cm2	680 – 750			1	
	PC	775	7.9 kW/cm2	-	30.8	57	4	

Supplementary Note 1. Blue-shift photoconversion *vs* red-shift photoconversion.

The contrast of photoconversion, calculated as the ratio of fluorescence in the detection channel of the photoconverted species after and before photoconversion, is strictly related to the magnitude of the spectral separation and the direction of photoconversion. Let us consider two forms of a protein, with absorption and emission bands spectrally shifted with respect to each other. The emission band used to collect the fluorescence of the red-shifted form has a higher degree of overlap with the fluorescence of the blue-shifted form than vice versa.

Therefore, if the photoconversion proceeds from blue to red, the contrast of photoconversion for the new form strictly depends on the possibility of using a wavelength of excitation exclusive to the red-shifted form. This condition can be satisfied in the green-to-red PCFPs of the GFP family where the spectral separation is around 100 nm (Supplementary Fig. 1a). It is important to underline the effect that this might have in the assessment of the fold of conversion for the given PCFP. When calculating the ratio of the fluorescence before and after PC (F^{postPC}/F^{prePC}) the denominator is close to zero, predominantly affected by the background of the detector. The value will therefore be mostly influenced by the fluorescence level of the photoconverted species, in turn defined by the specific microscope acquisition settings.

For the miRFPs with a smaller spectral gap (around 50–60 nm), the hypsochromic shift from red to blue still allows an efficient contrast for the photoconversion when measured as the change of fluorescence in the emission band of the new form (Supplementary Fig. 1b). The proximity between the two set of spectra makes the contrast of photoconversion in the channel of the original form more dependent on the excitation wavelength. A too blue-shifted wavelength will excite both the initial and the photoconverted form (Supplementary Fig. 24e). Furthermore, in the attempt of integrating the majority of the photoconverted form crosstalk with the ground form can arise reducing the contrast of photoconversion in the channel of the converted form. The magnitude of this influence will be linked not only to the extent of the emission band set, but also to the disparity in the brightness of the two forms.

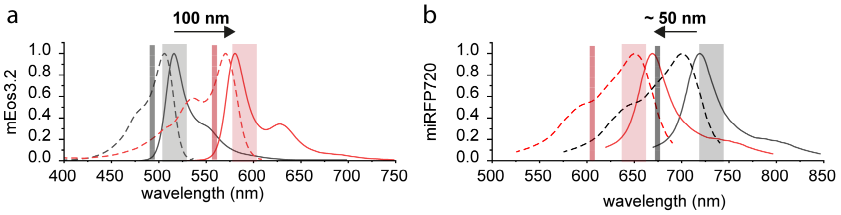

Supplementary Figure 1. Spectra of representative photoconversion mechanisms. The absorption (dotted lines) and emission (solid line) for the initial form (black line) and the photoconverted form (red line) of the protein are shown. The vertical lines identify the excitation wavelengths, while the vertical bands locate the detection window used in the experiments, both with the same color code for initial (black) and photoconverted form (red) (a) mEoS3.2 as an example of red-to-green PCFPs. Excitation lines at 488 nm and 560 nm. (b) miRFP720 as an example of NIR-to-far-red PCFPs. To have a more straightforward representation, the spectra of the non-photoconverted form have been shifted 50 nm to the left. Excitation lines at 610 nm and 670 nm.

Supplementary Note 2. Spectral and lifetime information in the photoconversion.

For the miRFP variants studied, the blue shift of the absorption and emission spectra is coupled with a change in lifetime (Supplementary Fig. 2). For the miRFP703, miRFP713, and miRFP720, with an average fluorescence lifetime of 0.6–0.8 ns for the NIR form (collected in the red-shifted detection window), the lifetime in the far-red form (collected in the blue-shifted detection window) is slower with an average fluorescence lifetime of 1–1.3 ns after photoconversion with 405 nm light at around 3–5 J/cm^2 (Supplementary Fig. 2a, c, d). The fluorescence lifetime for miRFP670

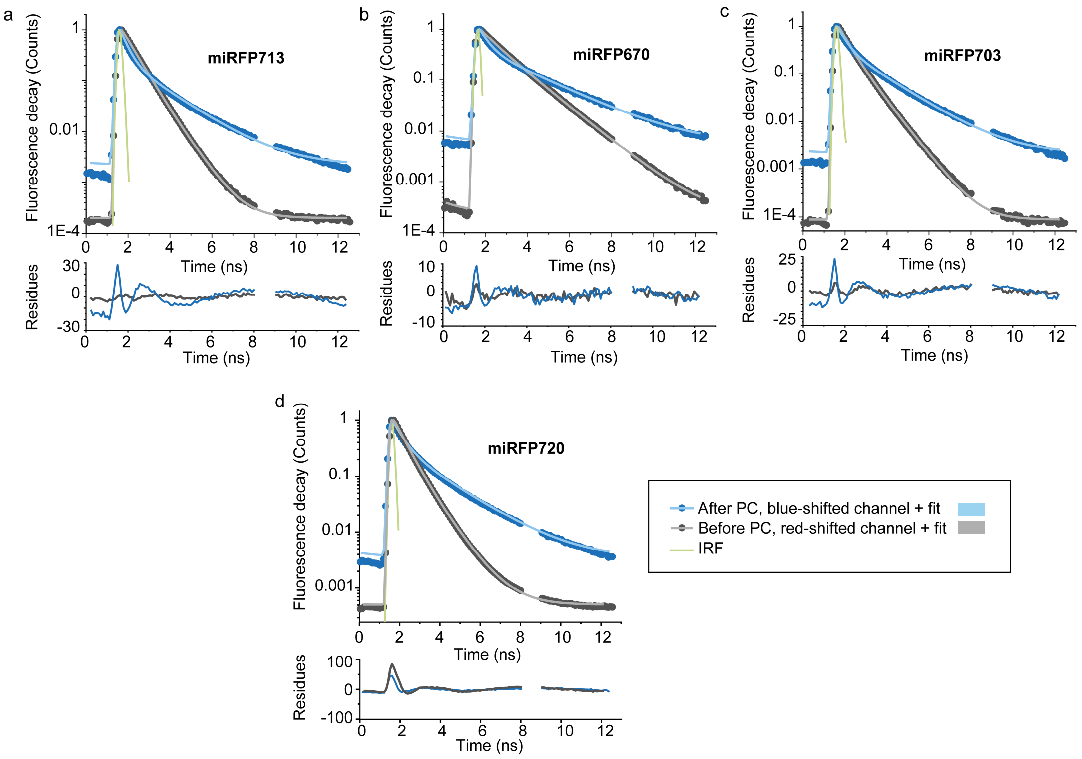

Supplementary Figure 2. Lifetime characterization of the miRFP. (a–d) FLIM images of *E. coli* expressing the miRFP of interest were collected before and after illumination with 405 nm light (~ 2–5 J/cm^2) and the fluorescence lifetime decay were extracted and analyzed, for (a) miRFP713, (b) miRFP670, (c) miRFP703, and (d) miRFP720. Fluorescence decay lifetime for the two characteristic channels of photoconversion, i.e. the red-shifted (detection band, 660–700 nm for miRFP670, 690–730 nm for miRFP703, 700–740 nm for miRFP713, 710–750 nm for miRFP720, gray dots) and the blue-shifted channel (detection band, 570–600 nm for miRFP670, 600–630 nm for miRFP703, 610–640 nm for miRFP713, 620–650 nm for miRFP720, blue dots) are reported. The IRF of the system is outlined as the solid light green line. For both forms a two-component decay has been considered for the fitting (solid lines). For example, for miRFP720, the decay of the NIR form (gray line) has a mean lifetime of 0.686 ± 0.001 ns (with 79% τ_1 = 0.56 ± 0.01 and 21% τ_2 = 0.96 ± 0.06 ns), while for the far-red form (blue line) the mean lifetime is 1.37 ± 0.04 ns (with 69% τ_1 = 0.49 ± 0.01 and 31% τ_2 = 1.87 ± 0.07 ns). Values and associated errors are the ones of the fit. The residues for the two fits are reported on the bottom following the same color code. The curves are representative examples of the 3–5 repetitions that are collectively reported in Fig. 2a.

instead goes from 1.2 ns to 1.8–2 ns after photoconversion, as measured in the red-shifted and blue-shifted detection windows, respectively (Supplementary Fig. 2b).

To further investigate the link between the lifetime and the photoconversion mechanism, we recorded the lifetime information spectrally under illumination with 594 nm light. A region of ~ 29 × 29 μm^2 of *E. coli* expressing miRFP720 was photoconverted at 5 J/cm^2 of 405 nm light, and fluorescence lifetime data was recorded in five spectral windows of 20 nm in width in the range of 640–740 nm. The fluorescence intensity from inside and outside of the photoconverted area is estimated by averaging the central region and the border, respectively, of a line profile of 11.35 μm width (Supplementary Fig. 3a and yellow box (Supplementary Fig. 3e)). The fluorescence from the photoconverted region peaks at 660–680 nm, while the non-photoconverted area has a maximum in the 700–720 nm spectral band (Supplementary Fig. 3b, mean ± std is reported in the graph). The ratio between the photoconverted and non-photoconverted mean (i.e. the yield of photoconversion) decreases from 640 to 740 nm due to the relative population and spectral profile

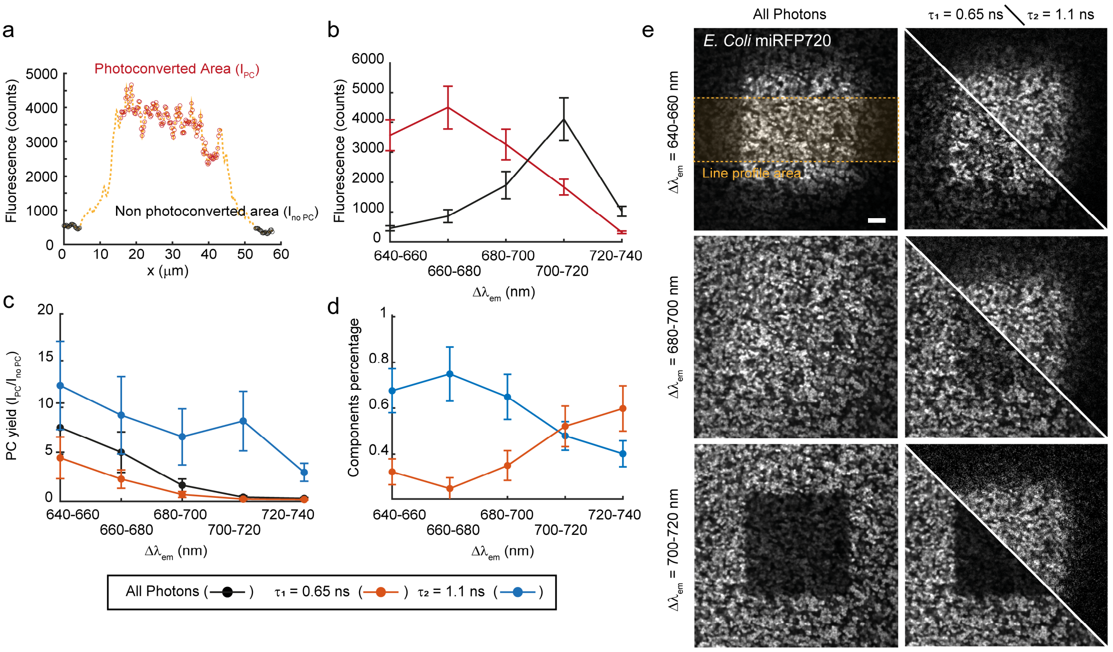

Supplementary Figure 3. Lifetime dual components decomposition. (a) Line profile averaged over 11.35 μm (as illustrated in the first image of panel e). The red circles correspond to the photoconverted area, while the black circle the non-photoconverted area over which the intensity is averaged to estimate the yield of conversion as well as the intensity dependence to the spectral band (mean and std of the following panels). (b) Fluorescence for the photoconverted (red) and non-photoconverted (black) region. (c) Yield of conversion calculated as the fluorescence in and outside of the photoconverted region. The black line refers to the raw image where all the photons are integrated without temporal tag. The blue and orange lines are the yield of conversion for the images resulting from the lifetime separation for the two lifetimes 0.65 ns and 1.1 ns. (d) Amplitudes of the two components. (e) Examples of images for all the photons (left column) and for the two components (right columns) at three representative emission intervals: blue-shifted (up), red-shifted (bottom), and equilibrium between the two (middle). Scale bar, 5 μm.

of the two forms (solid black line, Supplementary Fig. 3c).

The fluorescence lifetime data is then fitted with a double exponential decay, fixing the decay time to the values recorded for the photoconverted (1.1 ns) and non-photoconverted (0.65 ns) miRFP720 (Supplementary Fig. 3d). Examples of the two resulting components of the fit and the original raw images are shown in Supplementary Fig. 3e (right and left column, respectively). The relative amplitude for the two components depends on the spectral window (Supplementary Fig. 3d), with the fast lifetime component being predominant at longer wavelengths, typical of the non-photoconverted form. The conversion yield calculated in the slow component is twice higher with respect to the raw image, while it is lower for the image of the fast component (Supplementary Fig. 3c).

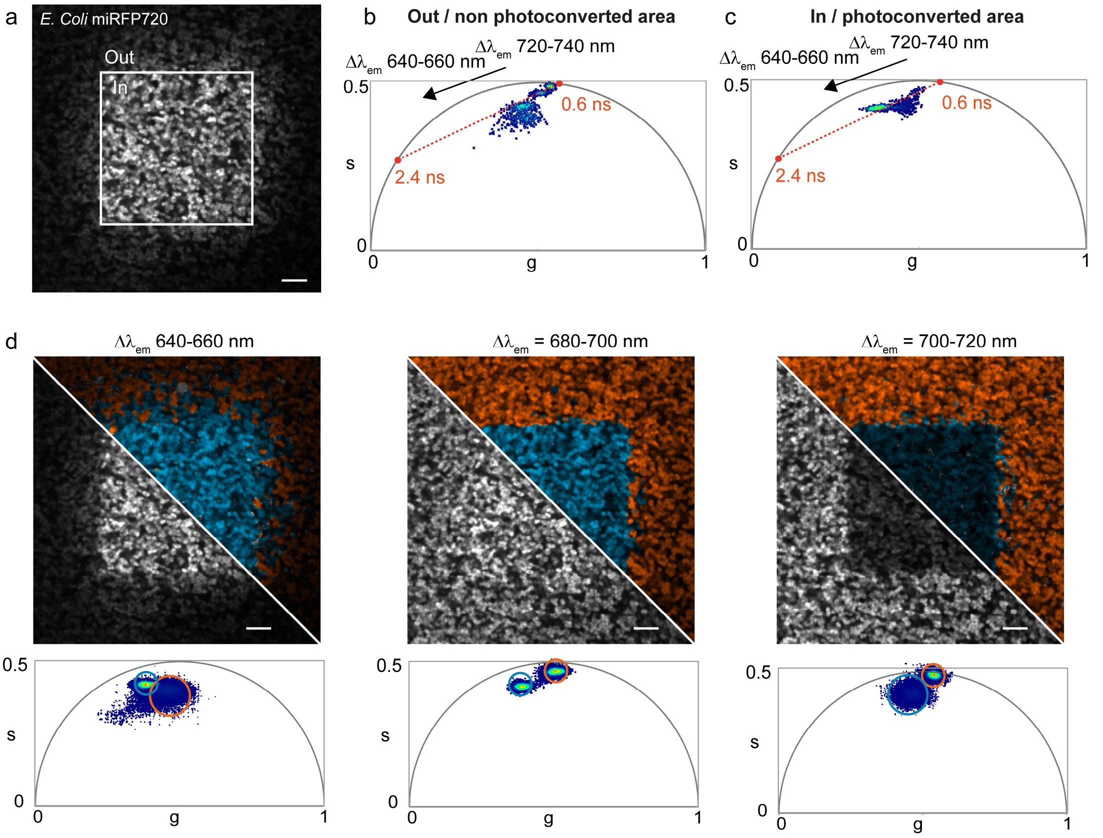

Supplementary Figure 4. Spectral lifetime exploration for miRFP720 in *E. Coli*. (a–c) Representation of FLIM data in phasor coordinates for the outside-box/non-photoconverted and inside-box/photoconverted region. The phasor plot has been calculated on an 8-pixel averaged image and all the spectral windows are reported as summed up in b and c. The arrows guide on the directionality with which the population moves starting from the red-shifted to the more blue-shifted wavelengths. The corresponding lifetime values on the semicircle are reported as reference points. (d) Example of the data in single emission bands. In the top row, the raw images (bottom left half) and the areas segmented from the phasor plots (top right half) are reported. In the bottom row, the relative phasor plots and the regions used for the segmentation are highlighted, at a binning of 2 pixels and a median filter of 5 pixels. Scale bars, 5 µm. The images are representative examples of 3 independent experiments with similar results.

Instead of fitting the fluorescence decay, the lifetime information in the image can be explored in a phasor plot (Supplementary Fig. 4). For the non-photoconverted miRFP720 (Supplementary Fig. 4a, outside box) the spectral distribution follows a line, moving from a single component of 0.6 ns to a combination of multiple components (Supplementary Fig. 4b). This behavior suggests a complexity where two components are, already at equilibrium, contributing to the fluorescence. In the photoactivated region (Supplementary Fig. 4a, inside box), the spectral dependency is less clear (Supplementary Fig. 4c). In the phasor plots in Supplementary Fig. 4b–c, all the images along the five spectral windows are summed up. Considering each spectral band separately, the areas in the phasor plot dominated by the photoconverted and non-photoconverted miRFP720 respectively results to be well separated and allows to segment and retrieve the information of the two spatially distinctive areas also in regions and spectral detection windows where purely spectral information will preclude it (Supplementary Fig. 4d).

Supplementary Note 3. Multiplexing strategies: green-to-red and NIR-to-far-red PCFPs.

The possibility of multiplexing photoconversion experiments depends on the ability to distinguish multiple proteins. If we limit the consideration only to the spectral characteristics, the NIR-to-far-red PCFPs are potentially compatible with both blue-to-green and green-to-red PCFPs. The orange-to-far-red would converge after photoconversion in the same spectral window as the NIR-to-far-red PCFPs, making it challenging to distinguish the two proteins. In particular, among the compatible groups of PCFPs, the green-to-red PCFPs are very attractive, both given the numerous variants available and their compatibility with single-molecule approaches.

Depending on the specific protein and the relative level of expression, the fluorescence tail of the photoconverted form of the green-to-red PCFPs can interfere with the detection of the photoconverted form of the NIR-to-far-red PCFPs (Supplementary Fig. 5). Indeed, when the photoconverted forms are simultaneously excited and recorded the fluorescence of mEos3.2

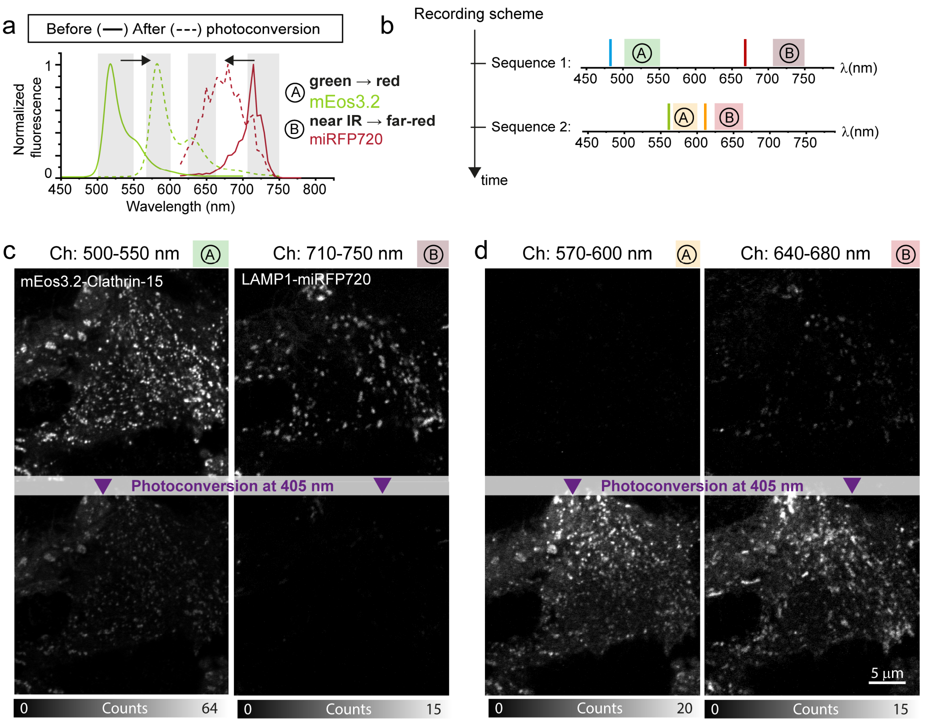

Supplementary Figure 5. Spectral compatibility of green-to-red and NIR-to-far-red with simultaneous recording. (a) Emission spectra for mEos3.2 (green) and miRFP720 (red) before (solid lines) and after (dotted lines) photoconversion, and the detection bandwidths (gray bands) used: 500–550 nm and 570–600 nm for the green-to-red mEos3.2, and 710–750 nm and 640–680 nm for the NIR-to-far-red miRFP720. (b) Sequence of acquisition for the reported images. In the first sequence the pre-photoconverted forms of the two proteins are recorded simultaneously and in the following the post-converted forms are recorded simultaneously. (c–d) Representative images from this recording scheme. The spectral vicinity and unbalanced brightness level of the post-photoconverted forms (d, bottom row), translate in a big bleed-through of the red form of mEos3.2 (left) into the far-red channel of the miRFP720 (right).

(considered here as an example of green-to-red PCFPs) is spectrally overlapping with the miRFP720 fluorescence, impairing a visual separation of the two structures.

The first strategy to minimize this bleed-through is to design a sequential illumination strategy (Supplementary Fig. 6). Splitting the excitation of the two PCFPs in time helps to recover a clear and selective visualization of either one or the other structure.

When the contribution of the two proteins in the various detection channels can be calibrated, i.e. they are labeling distinct structures in the cell with non-overlapping regions, it is possible to spectrally unmix the information to recover the separation between the two structures in a simultaneous recording (Supplementary Fig. 7).

For the reported images, the ImageJ plug-in "Spectral Unmixing" (Joachim Walter, https://imagej.nih.gov/ij/plugins/spectral-unmixing.html) has been used for this purpose. This

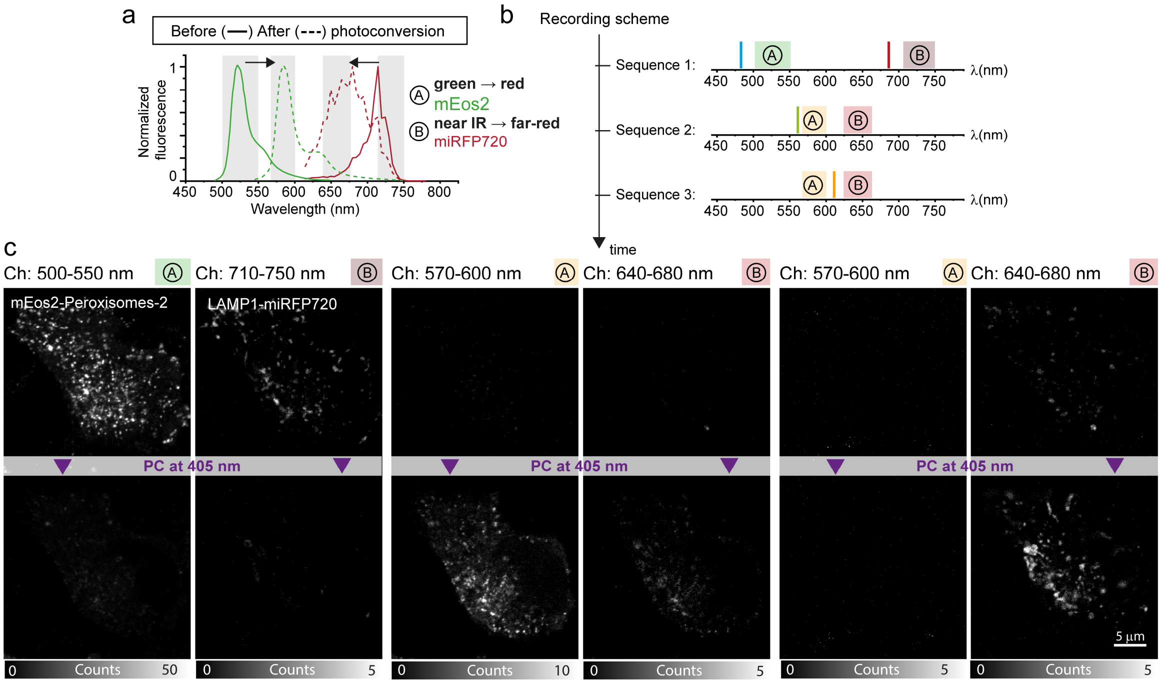

Supplementary Figure 6. Spectral compatibility of green-to-red and NIR-to-far-red PCFPs with sequential recording. (a) Emission spectra for mEos2 (green) and miRFP720 (red) before (solid lines) and after (dotted lines) photoconversion, and the detection bandwidths (gray bands) used: 500–550 nm and 570–600 nm for mEos2, and 710–750 nm to 640–680 nm for miRFP720. (b) The sequential recording is composed of three sequences: (1) simultaneous recording of the pre-photoconverted forms given their clear spectral separation, (2) excitation at 561 nm and recording in both channels of the post-photoconverted forms (570–600 nm and 640–680 nm), and (3) excitation at 610 nm and recording in both channels of the post-photoconverted forms (570–600 nm and 640–680 nm). (c) Example of this scheme of recording, with mEos2 tagged to peroxisomes (mEos2-Peroxisomes-2) and miRFP720 to lysosomes (LAMP1-miRFP720). The sequential recording strategy reduces the amount of bleed-through allowing for a clear separation of the two post-photoconverted forms (bottom row, third and last images from the left).

strategy can be performed only if the assumption of simultaneous recording for the two channels is respected. For a scanning approach, this is generally valid if the different channels are acquired on a line-by-line or pixel-by-pixel basis, while in a frame-by-frame recording the time between the recording of the different channels can invalidate the assumption of simultaneous recording, especially when fast-moving structures or organelles are imaged.

Lifetime is an additional dimension that can be explored to enhance the contrast in multiplexing experiments. miRFP720 has a characteristic lifetime (around 0.65 ns) that, upon photoconversion, increases but stays below 1.5 ns (Supplementary Fig. 2). Both values are faster than common PCFPs like Dendra2 or mEos3.2, characterized by lifetimes of around 2.5–3.5 ns (Supplementary Fig. 21) that also increase upon photoconversion. In a FLIM image, the two proteins are distinguishable, enabling a separation method compatible with a scenario where spectral properties are challenging to measure (Supplementary Fig. 8).

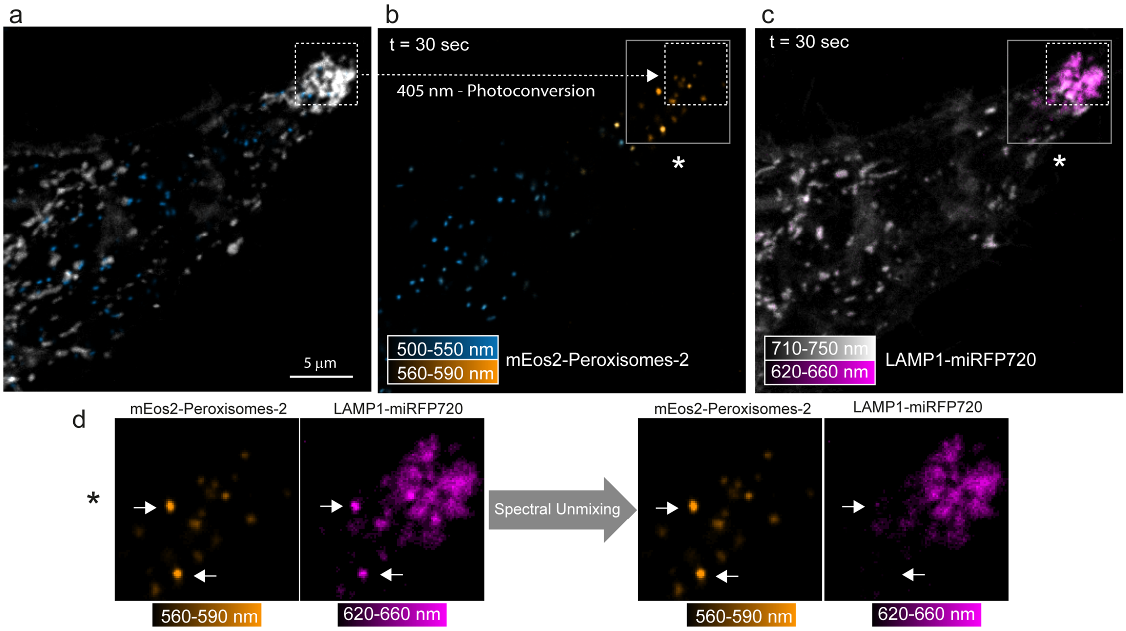

Supplementary Figure 7. Spectral unmixing of PCFPs. Imaging of miRFP720 tagged to lysosomes (LAMP1-miRFP720) and mEos2 to peroxisomes (mEos2-Peroxisomes-2). (a) Before photoconversion the signal is predominantly in the green for mEos2 (500–550 nm, cyan) and in the NIR for miRFP720 (710–750 nm, grey). (b–c) After photoconversion with 405 nm in the white dotted rectangle, the red form of mEos2 (560–590 nm, orange) and the far-red form of miRFP720 (620–660 nm, magenta) appears. The four channels are recorded line-by-line sequentially, and therefore they report the same instant in time. This temporal consistency and the possibility to calibrate the contribution of each species in the two detection bands for the photoconverted species (560–590 nm and 620–660 nm), allows to use a spectral unmixing algorithm to fully separate the two forms. (d) Zoom-in for the squared region indicated with the asterisk in b and c. Red and far-red channels showing the photoconverted forms are reported before (left) and after (right) spectral unmixing. The arrows specifically point to peroxisomes that can be seen in both channels before spectral unmixing and only in the red channel after.

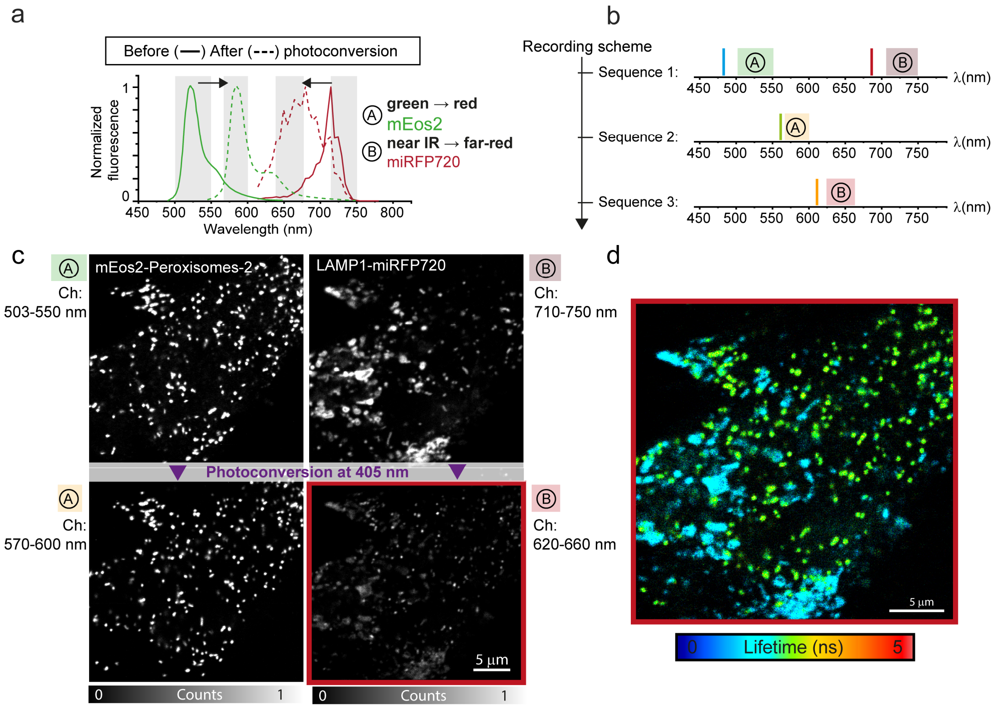

Supplementary Figure 8. Lifetime unmixing of PCFPs in FLIM imaging. (a) Excitation spectra for miRFP720 and mEos2 before (solid lines) and after (dotted lines) photoconversion. (b) Sequential recording scheme used for the imaging. (c) Example of such a recording where the channels indicated next to the images are reported, from a sample with miRFP720 labelling lysosomes (LAMP1-miRFP720) and mEos2 labelling peroxisomes (mEos2-Peroxisomes-2). The images are recorded frame-by-frame. Due to the properties of the labelling and of the PCFPs, residual bleed-through can appear also in a sequential scheme of recording. The difference in lifetime between the different PCFPs can be used to unmix the signal in the far-red channel to separate the contribution of the red form of mEos2 from the far-red form of the miRFP720. (d) Example of a FLIM image where the lifetime is reported per pixel. mEos2, labelling peroxisomes, can be clearly distinguished thanks to their longer lifetime (around 2.5 ns) from to miRFP720, labelling lysosomes (closer to 1 ns).

Supplementary Note 4. Role of the STED beam in the miRFP photoconversion.

We can simplify the interaction between the STED beam applied during STED imaging and the miRFP photoconversion by decoupling the two phenomena, first the photoconversion effect and then the STED imaging, and integrating them into the image scanning approach.

The 775 nm light has a spatially extended shape with minima and maxima of intensity (the donut shape reported in Supplementary Fig. 9a). When recording an image, the depletion beam and the excitation beam are overlapped and scanned over the field of view, sequentially centered on each part of the image in a pixel-by-pixel fashion. Therefore, each area of the sample, before being in the center of the STED beam, will be gradually exposed to an increasing light level up to the maxima of the spatially extended donut light pattern, and then a decreasing light level to the center of the STED beam (Supplementary Fig. 9a). If we sum the energy delivered to one pixel while the donut (of a commonly used STED illumination intensity) scans through, its value early on exceeds

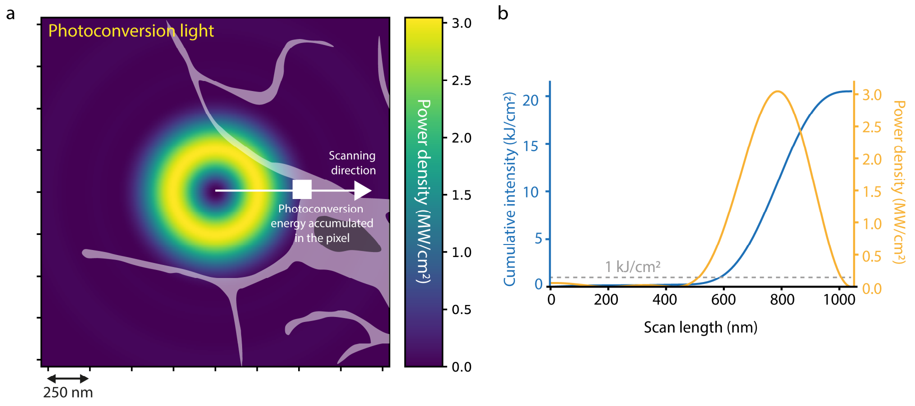

Supplementary Figure 9. Photoconversion induced by the STED beam. (a) Representation of the STED beam at 775 nm during the scanning process that forms the image acquisition. A region of the sample will be exposed to all the light of one side of the crest of the depletion beam light pattern before ending up in the center of the donut where the photons will finally be registered. The donut is calculated for an intensity of the 775 nm light at the back aperture of 15 mW. (b) Building up of the cumulative intensity (blue line) based on the previously described scanning of the STED beam (of which the line profile across half of it is reported as the yellow line). Noted is also the energy threshold for the photoconversion to take place at a plateau level (dashed gray line).

the threshold of around 1 kJ/cm^2 that corresponds to the plateau level for photoconversion with 775 nm light, as estimated by a homogeneous illumination (Supplementary Fig. 9b). From this calculation, we can deduce that the majority of the miRFP population has been converted to the blue-shifted form before the STED beam is centered on the pixel. Therefore, the photoconverted proteins will show up mainly in the blue-shifted image when the pixel is recorded. While this estimation only takes the scanning of a single line into account, in scanning a 2D image there will be further illumination intensity added up to each pixel from the scanning of previous lines, as the donut pattern is isotropically distributed along the fast as the slow scanning axis.

Starting from this unbalance between the two forms imprinted by the scanning of the STED beam, STED imaging has been performed simultaneously in the two channels, using a common 640 nm

excitation beam and two spectrally separated detection windows (blue-shifted, 650 – 690 nm, and red-shifted, 705 – 745 nm). In the two detection there is a higher signal-to-noise ratio in the blue-shifted channel with respect to the red-shifted channel as quantified in Fig. 5c). Although we do not exclude a possible non-trivial interaction between the stimulated emission depletion and photoconversion, the recorded data does not point to any mixed effect of both processes applied simultaneously. Further spectroscopic studies might help decouple the nature and competition of the two processes more in detail.

Supplementary Note 5. Phototoxicity assessment.

Photoconversion mechanisms provide a functional readout but intrinsically demand relatively higher illumination conditions, as additional light needs to be delivered to the sample to trigger the photoconversion. Here we compare the microscope acquisition settings (illumination power, pixel dwell times, repetitions) (1) required by miRFPs with the different investigated photoconversion-inducing illumination conditions (wavelength, pulse width, repetition rate), with either (2) applications that work with the same illumination conditions, or (3) photoconversion mechanisms in other families of PCFPs

Supplementary Table 2. Energy and power level for the photoconversion light sources.

λ^{PC}	**Energy at saturation for miRFP**	**<P>***	p_{peak}*	**Irradiance limit**
UV/Vis CW: 405 nm	~ 5 J/cm^2	~ 2 mW	- †	~50 J/cm^2
NIR 550 ps: 775 nm	200 J/cm^2	~15 mW	~ 680 mW	< 10 mW **
NIR 120 fs: 670 – 800 nm	4 J/cm^2	~ 4 mW	~500 W	~1 kJ/cm^2 or 7 – 10 mW**

* Average power, **<P>**, and peak power, p_{peak}, at the back aperture of the objective.
** Average power at the sample plane
† Note that for the CW laser the two values of power correspond.

a. UV/Vis continuous-wave light. For 405 nm light, the energy required for photoconversion of miRFPs is ~5 J/cm^2. As a key wavelength of many photoswitchable FPs the photodamage induced by the UV light has been extensively characterized in the literature, in particular Waldchen et al[1], quantified the photoresistance of transfected cells upon irradiation at different wavelength using cell division as the assessment parameter.

They characterized how the phototoxicity increases with decreasing wavelengths, identifying the limit of ~50 J/cm^2 for 405 nm irradiation and ~1 kJ/cm^2 when moving toward the NIR (their experimental setting stop at 640 nm). In our photoconversion studies we are within the limit defined by Waldchen et al at 405 nm with the ability of tuning the phototoxicity effect by choosing the most NIR shifted wavelengths for photoconversion. Nevertheless, careful considerations need always to be done when assessing phototoxicity, not only on the wavelength but also cell line, phase of the cell and illumination conditions.

b. NIR pulsed light with a 550 ps pulse width and 40 MHz repetition rate. Similar considerations can be drawn for the broader 775 nm laser pulse. This laser source is typically used in STED microscopes as a depletion beam. Also, for this technique, *in vivo* imaging and phototoxicity have been extensively characterized[2] and the reported energy doses for STED imaging[3] (40 to 370 kJ/cm^2) is one-to-two orders of magnitude higher than that required for photoconversion of miRFPs (< 1 kJ/cm^2).

c. <u>NIR pulsed light with a 120 ps pulse width and 80 MHz repetition rate.</u> This laser source is commonly used for two-photon imaging and the power density required by miRFP photoconversion is comparable to the values reported in two-photon *in vivo* application. At 700

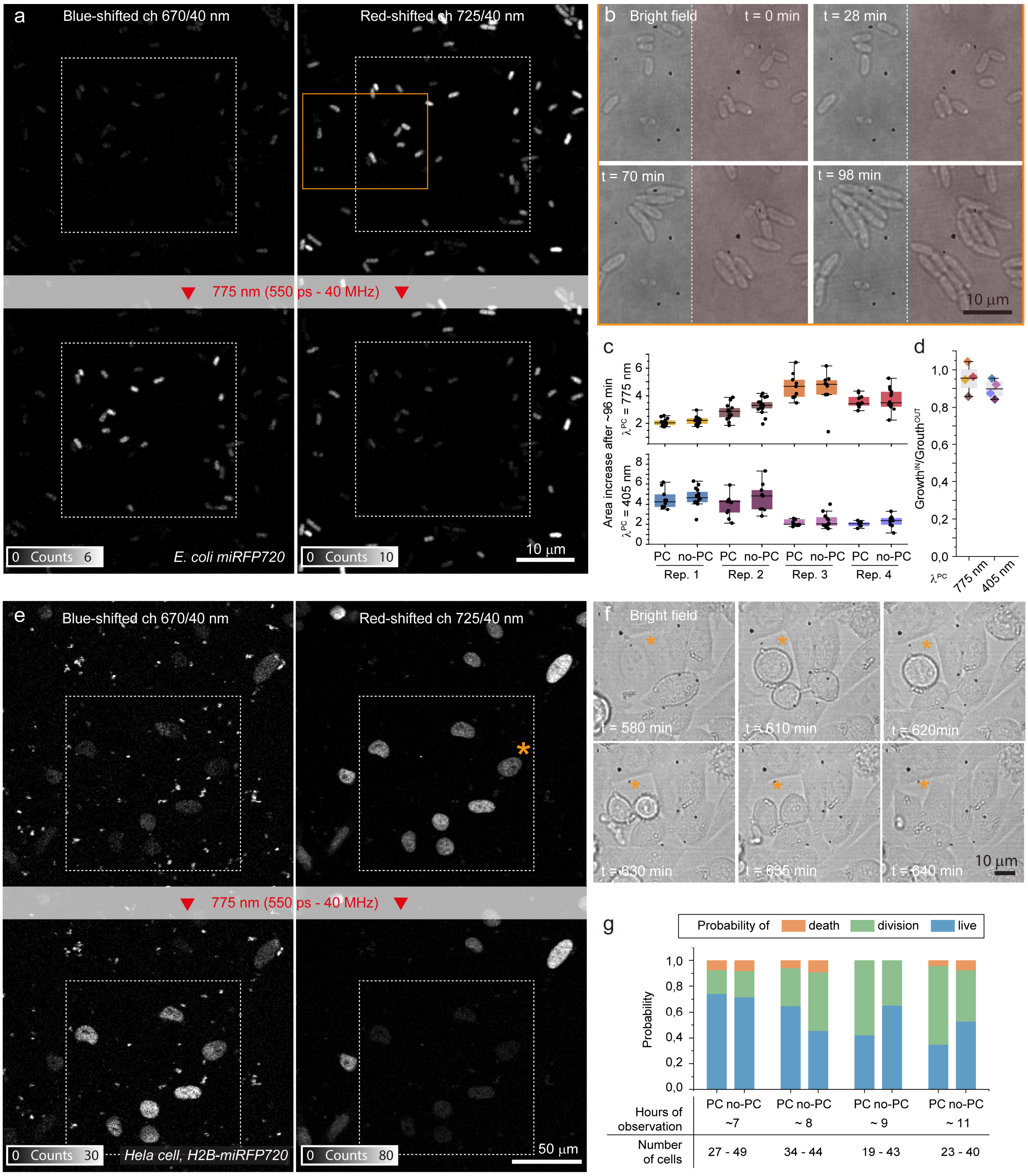

Supplementary Figure 10. Cell viability after photoconversion. (a) *E. coli* expressing miRFP720 have been photoactivated with 775 nm light at saturation level in a region of around 25 µm (dotted square) and (b) subsequently followed in brightfield for around 100 min over a bigger region of 50 µm. This allows to follow at once the growth of bacteria in and out of the photoconverted region, quantifying the increase in area of a bacteria colony (c, top). The same experiment has been repeated for photoconversion with UV/Violet light at a power of saturation for Dendra2 (bottom). The box plot reports the ratio of the colony area in the last time point (~ 96 min) with respect to the first time point in four independent repetitions. The box indicates the 25 to 75% and the line shows the mean. (d) To directly compare the effect of the two wavelengths of photoconversion the ratio between the growth in the photoconverted area over that in the non-photoconverted area is reported, where the color code of each dataset matches between the two box plots. (e) Phototoxicity assay through cell division upon 775 nm illumination. Hela cells transiently expressing H2B-miRFP720 have been photoconverted in a confined area (dashed square) and (f) subsequently followed by monitoring a larger area in brightfield over a time period of 7–11 hours. (g) Each cell enclosed in the field of view has been labelled according to their fate over the observation period: death, division or unperturbed behavior ("live"). The probability of each fate is then reported in the stacked columns, together with the number of cells for each area (photoconverted and non-photoconverted) and the time of observation. To note is that the analysis report on all the cells, transfected and not.

nm we used around 4 mW at the back aperture with a dwell time of 3.16 µs and the reported values for two-photon imaging is between 1 and 50 mW[4]. More specifically, different systematic studies on photodamage for non-linear excitation identify ~ 7–10 mW as the limit average laser power on the specimen before critical cell death[5,6]. The compared studies rely on different assays to quantify damage: cell membrane integrity, cloning efficiency, viability measurements in different systems, and reactive oxygen species, and they all converge to the same limit. It is important to note that these earlier studies on phototoxicity always account for repetitive scanning (that would correspond to a timelapse acquisition, ~ 10 frames), while the photoconversion in our study is generally performed only for the number of times required to photoconvert the region of interest. Another aspect that often makes a comparison between different studies difficult is the dwell time used in the imaging, from a few µs[5] to tens of µs[6].

In addition to the literature references, we experimentally tested the effect on cell viability for the described blue-shift photoconversion mechanism and how it compares to the commonly used red-shift photoconversion mechanism, of which we considered Dendra2 as our standard.

We monitored the growth of miRFP720-expressing *E. coli* colonies over a period of around 100 min when illuminated or not by a photoconversion wavelength: either 775 nm (550 ps, 40 MHz) at the saturation level required by miRFP720 photoconversion, or 405 nm (CW) at the saturation level required by Dendra2 (Supplementary Figure 10a–c). The ratio of growth for the NIR-photoconverted and non-photoconverted area is close to 1, and similar to the ratio of growth obtained for the UV/Violet-photoconversion (Supplementary Figure 10d).

We also monitored Hela cells in a cell division assay over 7–11 h. Here, similarly, the probability of cell division for Hela cells does not show any change upon NIR illumination, neither is any increase of cell death observed (Supplementary Figure 10e–g). The probability of division in and out of the photoconverted area are both within the range expected if we consider a division time of 22 hours for Hela cells and no systematic difference is observed.

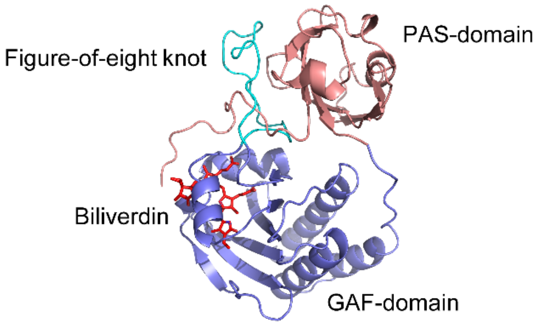

Supplementary Figure 11. Typical X-ray structure of an miRFP protein. Specifically, the structure of miRFP670 (PDB ID: 5VIV, https://doi.org/10.2210/pdb5VIV/pdb) is shown. The biliverdin chromophore is in red. The PAS and GAF domains are in pink and dark blue, respectively. The figure-of-eight knot extension of the GAF domain is in blue.

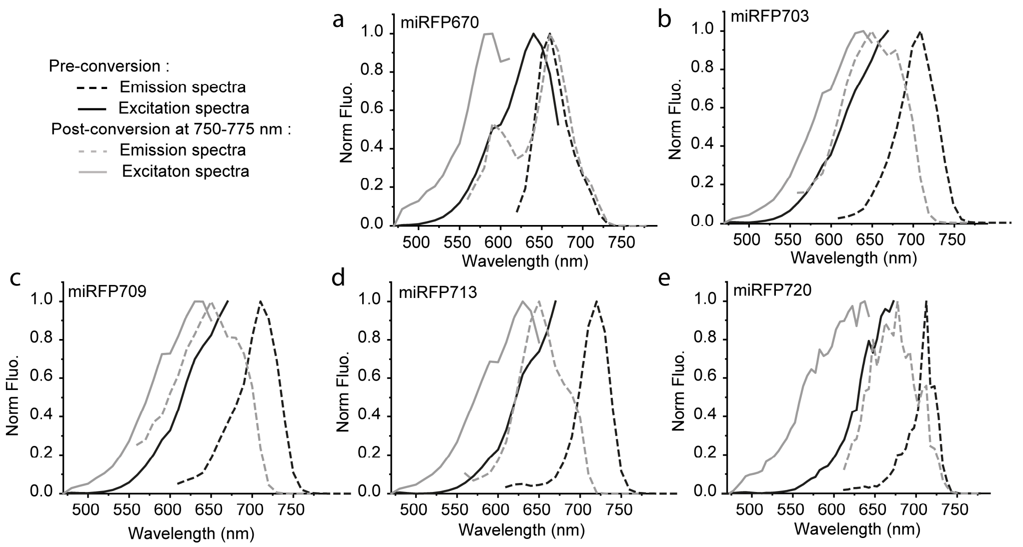

Supplementary Figure 12. Fluorescence spectra before and after photoconversion for different miRFP variants. The emission (dashed lines) and excitation (solid lines) spectra for (a) miRFP670, (b) miRFP703, (c) miRFP709, (d) miRFP713, and (e) miRFP720 have been recorded before (black) and after (gray) inducing the blue-shift photoconversion using a femtosecond Ti:Sapphire laser at 750 nm.

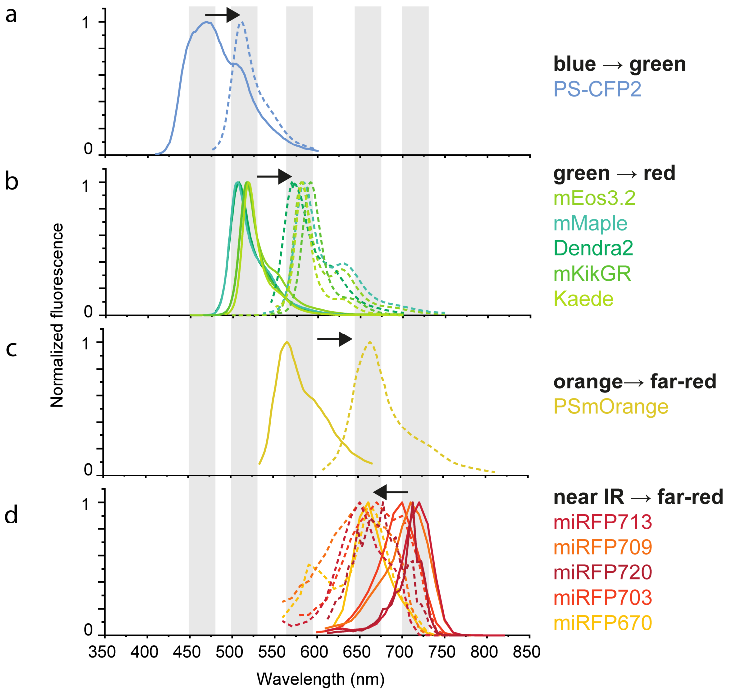

Supplementary Figure 13. Fluorescence shift for different PCFPs. It is possible to distinguish three main groups of GFP-like PCFPs, all with red-shifts to longer wavelengths, according to the portion of the spectra that they occupy: (a) blue-to-green: PS-CFP2[7], (b) green-to-red: mEos3.2[8], mMaple[9], Dendra2[10], mKikGR[11], Kaede[12], and (c) orange-to-far-red: PSmOrange[13]. The more numerous group is the green-to-red PCFPs. The different PCFPs occupy most of the visible spectra and are challenging to combine due to the overlap between the fluorescence of the photoconverted forms for one PCFP and the fluorescence of the ground forms for another PCFP. (d) miRFPs, that converts from NIR to far-red, and their photoconversion wavelengths make them optimal PCFPs for the far-red spectral region. The opposite direction of the shift, blue-shift to shorter wavelengths, has the advantage of not interfering with the fluorescence of GFP-like PCFPs. Spectra from FPbase[14].

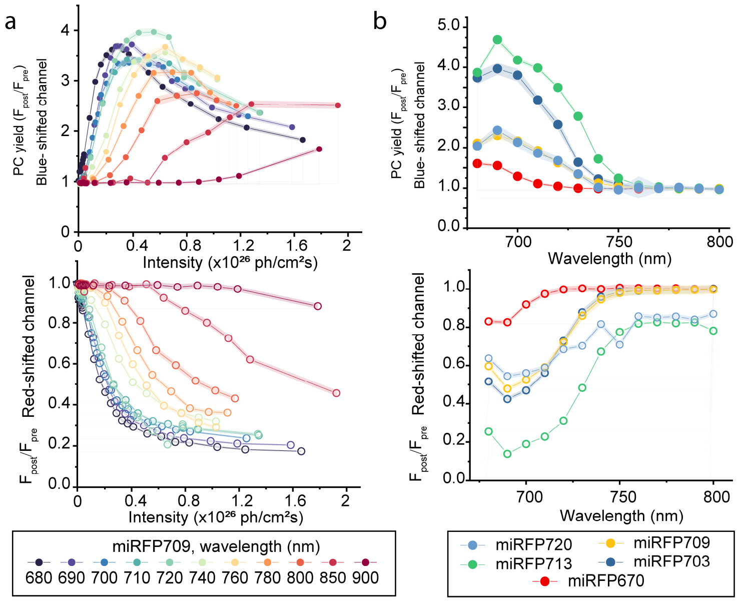

Supplementary Figure 14. Power and wavelength dependence of photoconversion with a Ti:Sapphire laser. (a) Photoconversion yield (measured as the ratio of the fluorescence before and after photoconversion, F_{post}/ F_{pre}) for miRFP709 at increasing light intensity and different wavelengths in the blue-shifted (filled circles, top panel) and red-shifted (empty circles, bottom panel) channels. (b) Action spectra for different miRFP proteins in the blue-shifted (filled circles, upper panel) and red-shifted (empty circles, lower panel) channels. Each data point is the mean for bacteria enclosed in a field of view of ~ 30 × 30 µm^2 (~ 200 bacteria). SD shown as the shaded areas around each point. The graphs show an extended intensity range compared to the corresponding panels in Figure 1.

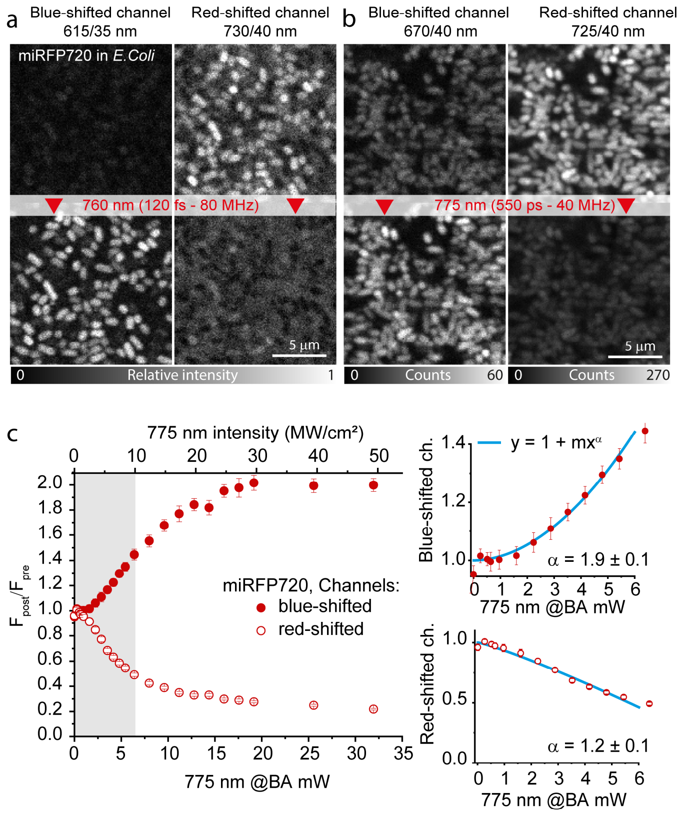

Supplementary Figure 15. Photoconversion of miRFP720 induced by NIR pulsed laser sources. (a) Representative images of an experimental recording with a 760 nm femtosecond pulsed laser as a photoconversion source. The detection windows and excitation wavelengths for the two channels are the same as in Fig. 1a. (b) Representative images of an experimental recording with a 775 nm picosecond pulsed laser (commonly used for depletion in STED imaging) as a photoconversion source. Experiment sequence for (a–b): upon excitation with 640 nm light, the fluorescence in the two detection channels (blue-shifted, 650–690 nm, and red-shifted, 705–745 nm) is recorded; after illumination of the same area with 775 nm light, the fluorescence in the two channels is once again recorded. There is a change of fluorescence in the two-channels, with an increase in the blue-shifted channel and a decrease in the red-shifted channel, indicating the photoconversion. (c) Photoconversion yield as a function of the 775 nm light intensity as measured at the back aperture of the objective lens (left). The red-shifted channel is represented as empty circles, and the blue-shifted channel as filled circles. Zoom-in to the beginning of the power dependence (grey shaded area) for the blue-shifted (right, top) and red-shifted channel (right, bottom). Both curves are fitted with a power equation $y = 1 + mx^{\alpha}$. Each data point is the mean, and error bars show the SD, for bacteria enclosed in a field of view of ~ 30 × 30 μm^2 (~ 200 bacteria).

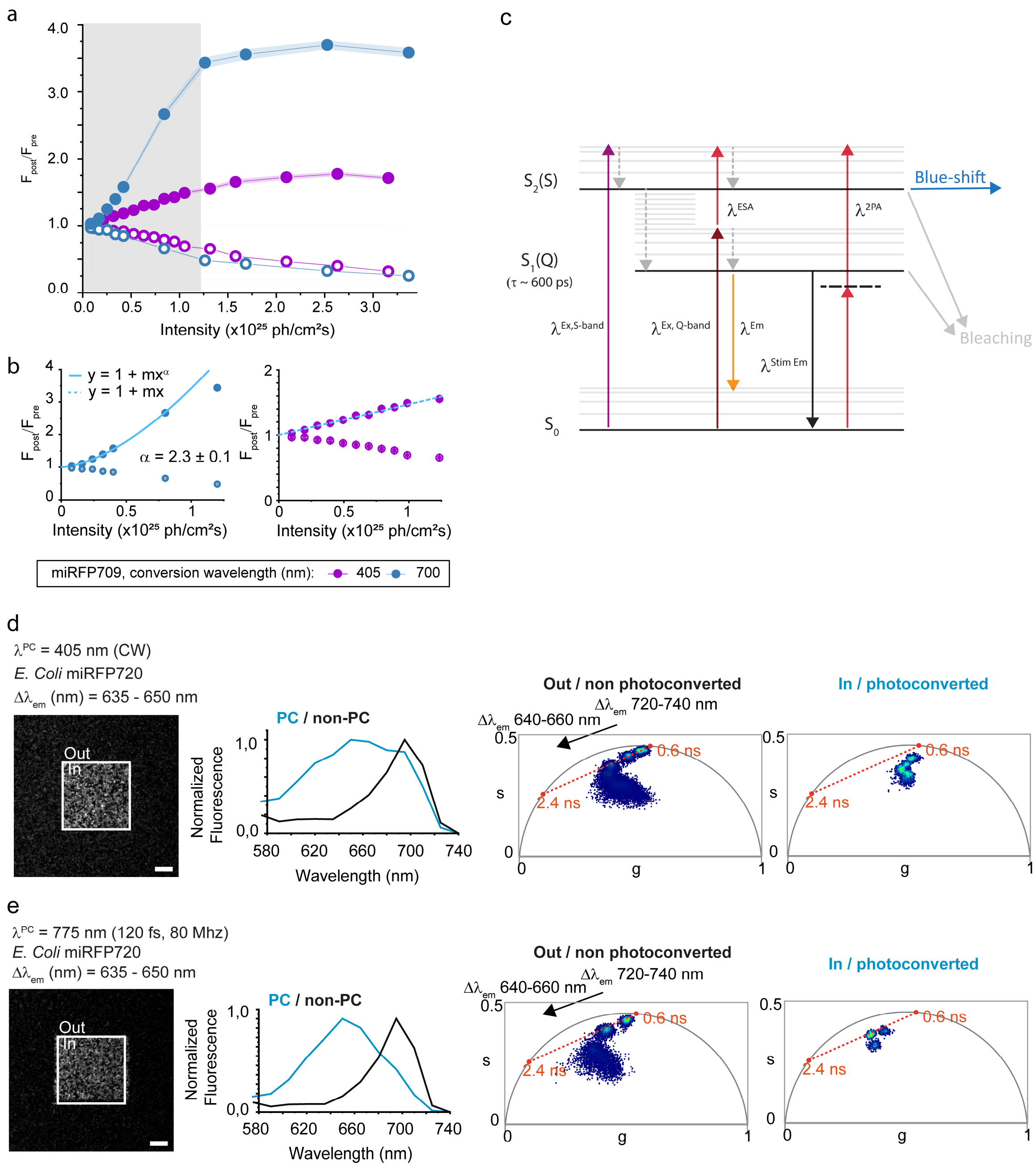

Supplementary Figure 16. Dependence of photoconversion on illumination intensity and wavelength. (a) Photoconversion yield at 700 nm (blue), absorbed by the Q band, and 405 nm (violet), absorbed by the Soret band, as dependent on the illumination intensity. The red-shifted channel is represented as empty circles, and the blue-shifted channel as filled circles. Each data point is the mean, and shaded areas show the SD, for bacteria enclosed in a field of view of ~ 30 × 30 µm^2 (~ 200 bacteria). (b) Zoom-in to the grey shaded area in (a) for the two wavelengths, 700 nm (left) and 405 nm (right). The degree of photoconversion in the blue-shifted channel is fitted with a power equation $y = 1 + mx^{\alpha}$ (700 nm, solid line) or a line $y = 1 + mx$ (405 nm, dashed line). (c) Scheme of the electronic transitions

that are suggested to underlie the photoconversion. (d–e) Comparison of lifetime and spectral changes after photoconversion triggered by 405 nm (d) and 775 nm (e) illumination. Scale bars, 5 µm. The photoconversion effects in the photoconverted areas (boxes) in the images (left) are reported both on the spectra (middle), as emission spectra before (black lines) and after (blue lines) photoconversion, and fluorescence lifetime (right), through the phasor plot obtained by summing up the multiple spectral windows between 640–740 nm, as indicated by the arrows above the plots. The photoconversion is done on *E. coli* expressing miRFP720.

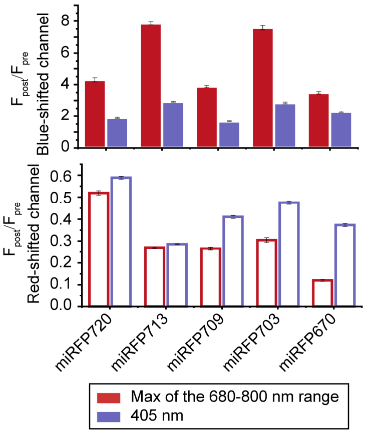

Supplementary Figure 17. Efficiencies of photoconversion for violet and NIR light for miRFP variants. Comparison of the maximum fold of conversion obtained for the different miRFP proteins either illuminated in the 700–750 nm region (red) or at 405 nm (violet). The filled bars (top) report the fluorescence increase in the blue-shifted channel upon photoconversion, while the empty bars (bottom) report the fluorescence decrease in the red-shifted channel upon photoconversion. Each data point is the mean, and error bars show SD, for bacteria enclosed in a field of view of ~ 30 × 30 μm^2 (~ 200 bacteria).

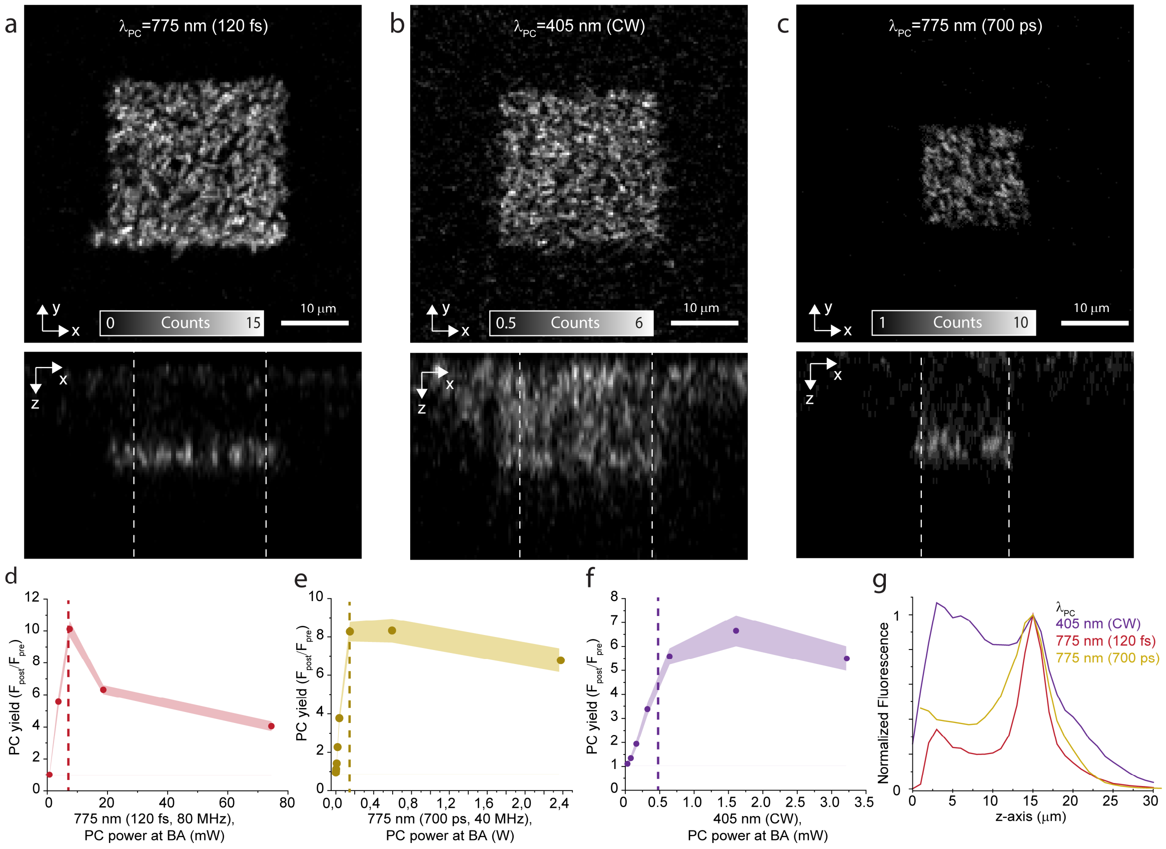

Supplementary Figure 18. Optical sectioning for different photoconversion wavelengths. *E. coli* expressing miRFP713 illuminated with different photoconversion-inducing illumination sources: 775 nm (120 fs, 80 MHz) (a), 405 nm (CW) (b), and 775 nm (700 ps, 40 MHz) (c). (a–c) The photoconverted area is at 15 μm from the cover glass, and in the images the blue-shifted channel after photoconversion is reported for the xy plane at z = 15 μm (up) and the xz projection (down). (d-e) Photoconversion yield for the blue-shifted channel at increasing laser power of the photoconverting-inducing illumination at 775 nm (120 fs, 80 MHz) (d), 775 nm (700 ps, 40 MHz) (e) or 405 nm (CW) (f). The vertical dotted lines specify the energy of the experiment in panels (a–b). It is important to note that these images have been acquired on a scanning system, therefore the effective energy per pixel is convoluted with the scanning. Each reported data point is the mean, and the shaded area is the SD, of the photoconversion yield for the bacteria enclosed in an area of 15 × 15 μm^2. (g) Intensity profile along the z-axis for the area enclosed in the dotted lines of panel a-to-c. All experiments have been repeated twice for each of the conditions.

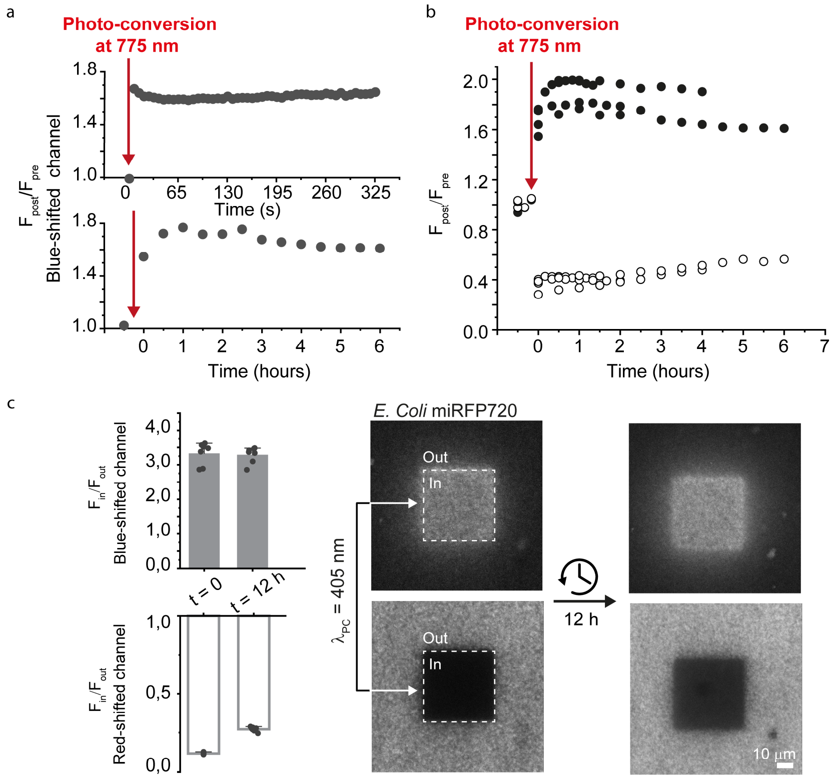

Supplementary Figure 19. Temporal stability of the photoconverted state at room temperature. (a) Stability of the photoconverted state at different time scales: seconds (top) and hours (bottom). The fluorescence in the blue-shifted channel is normalized to the intensity before photoconversion. (b) Repetitions of the thermal stability for the photoconversion with 775 nm light at 21 kW/cm^2 over a maximum of 6 h and at different time interval (10, 20, or 30 min) between frames. Fluorescence has been excited with a 640 nm intensity of 2.3 kW/cm^2. Reported is integrated intensity over the photoconverted area in blue-shifted (650–690 nm, filled circles) and red-shifted (705–745 nm, empty circles) channels, normalized to the intensity before photoconversion. (c) Long-term stability of the photoconversion investigated by observing the change in the ratio of conversion after 12 hours on a layer of *E. coli* expressing miRFP720 enclosed in a humidified chamber. The red-shifted species is recorded at 700–750 nm and excited at 633 nm (empty bars), while the blue-shifted species is recorded at 610–670 nm and excited at 561 nm (filled bars). The yield of conversion is calculated in the two time points separately, considering the ratio between the fluorescence inside and outside the area where the photoconversion has been induced. The use of a ratiometric approach allows to take into account variations in the sample that occur over the 12 hours of the measurements. N = 7 repetitions of which one representative image is reported. Bars show the mean value, and error bars show the SD.

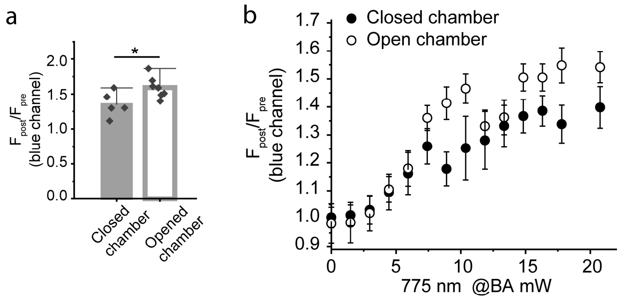

Supplementary Figure 20. Oxygen dependence of photoconversion. (a) The yield of photoconversion for bacteria expressing miRFP720 either closed in a closed chamber (low oxygen influx, filled bar) or placed in an open chamber (normal oxygen influx, empty bar). The bars show the mean value, and error bars show the SD, of N = 4-6 repetitions. Statistical test: two-sample Student's *t*-test, p=0.03. (b) The yield of the photoconversion as a function of photoconversion-inducing 775 nm illumination intensity for the two conditions of open (normal oxygen, empty dots) and sealed (low oxygen, filled dots). It is important to notice that other changes, like the pH value, might affect the analysis. Each data point shows the mean value, and error bars show SD, for bacteria enclosed in a field of view of ~ 50 × 50 μm^2 (~ 100 bacteria).

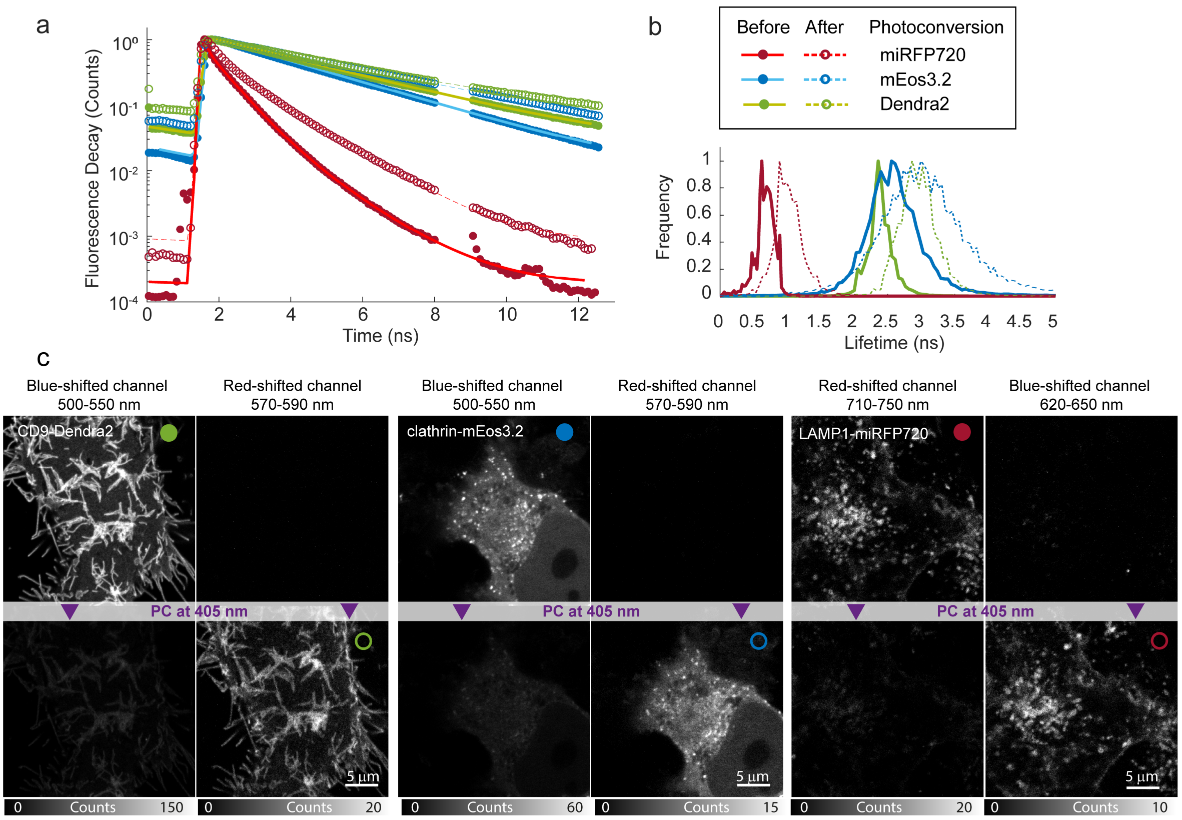

Supplementary figure 21. Fluorescence lifetime upon photoconversion in green-to-red and NIR-to-far-red PCFPs. (a) Fluorescence decays for miRFP720 (red), mEos3.2 (blue), and Dendra2 (green) before (filled dots) and after (empty dots) photoconversion. For all the proteins the photoconverted forms show a slower fluorescence decay. For mEos3.2 the lifetime changes from 2.71 ± 0.01 ns to 3.15 ± 0.01 ns, for Dendra2 from 2.65 ± 0.01 ns to 4.00 ± 0.01 ns, and for miRFP720 from 0.721 ± 0.001 ns to 1.05 ± 0.02 ns. The green-to-red PCFPs has been fitted with a single component, while two components are required to describe miRFP720. (b) Additional visualization of the same dataset, where the histogram of the fast FLIM lifetime is reported. (c) Images in the blue-shifted (left) and red-shifted (right) channels from which the lifetime has been calculated, before (top row) and after (bottom row) photoconversion, for the three fluorescent proteins and structures: CD9-Dendra2 (left), clathrin-mEos3.2 (middle), and LAMP1-miRFP720 (right).

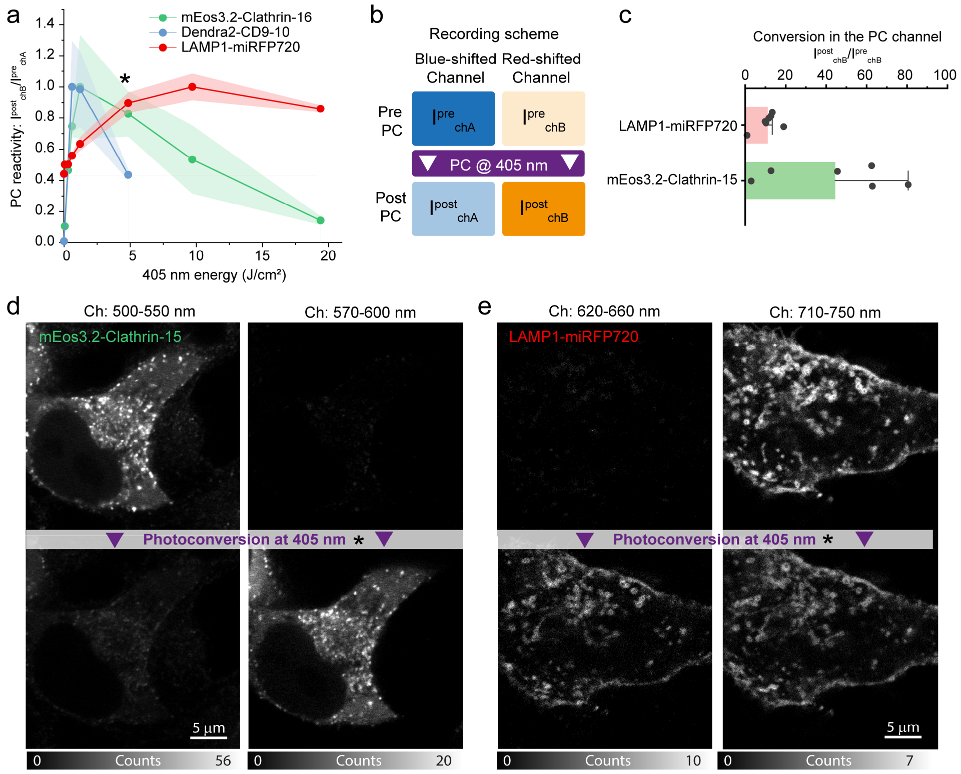

Supplementary figure 22. Photoconversion reactivity to 405 nm light for different PCFPs. (a) The reactivity to a 405 nm laser as photoconverting-inducing laser source is investigated for mEos3.2 (green), Dendra2 (blue), and miRFP720 (red), in cells where the protein is labelled to different structures; clathrin, CD9, and LAMP1, respectively. The reactivity is reported as the ratio between the fluorescence of the photoconverted form (I^{post}_{chB}) and the fluorescence in the starting condition (I^{pre}_{chA}). To compare the behavior of the different proteins the curves have been normalized to their maximum value. For all of them the 405 nm efficiently triggers photoconversion, until bleaching starts to prevail. Green-to-red PCFPs are more reactive to 405 nm light compared to miRFP720, but mEos3.2 and miRFP720 share a common 405 nm intensity range where both can be photoconverted with similar efficiencies (between 3–7 J/cm^2). The asterisk identifies the intensity of 405 nm used in the following panels to compare mEos3.2 and miRFP720, i.e. 4.8 J/cm^2. Each data point is the mean and shaded areas show the SD of the yield of conversion. (b) Recording scheme, where all images are recorded sequentially in time. (c) Yield of conversion for the photoconverted form of the protein for the same 405 nm energy (N ≥ 5 cells). (d–e) Representative images of photoconversion for both mEos3.2-Clathrin-16 (d) and LAMP1-miRFP720 (e), where the images are arranged in the same order of panel (b), where the top row is before photoconversion and the bottom row is after photoconversion, for the blue-shifted and red-shifted channels to the left and right respectively.

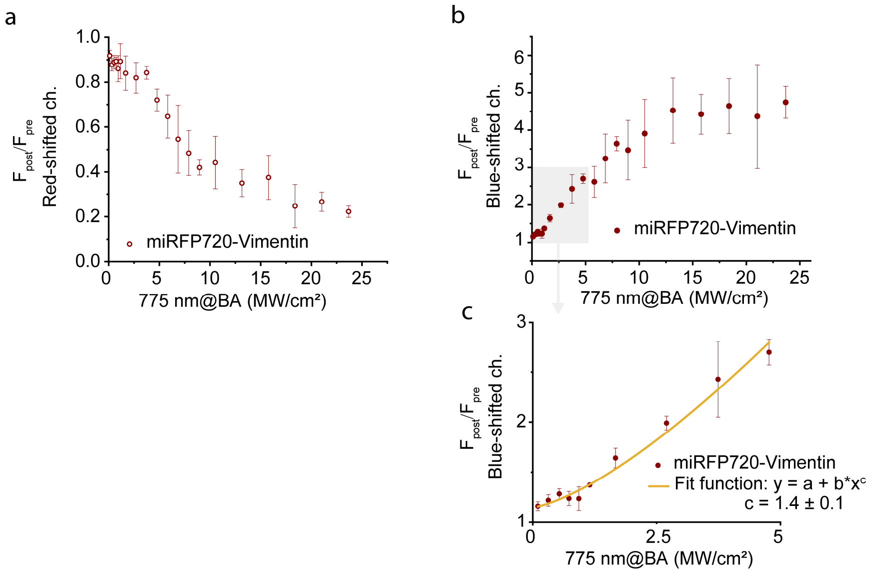

Supplementary Figure 23. Photoconversion of miRFP720 in mammalian cells induced by 775 nm. Photoconversion process for miRFP720-vimentin fusion protein measured with the sequence of three confocal images with excitation wavelengths 640 nm – 775 nm – 640 nm. The 775 nm power is varied up to 25 mW, while the 640 nm power is constant at 34.5 μW, and the dwell time is constant at 100 μs. (a) The decrease in the red-shifted channel (705 – 745 nm) shows two different slopes, where bleaching is a major contributor to the slope shift. (b) The photoconversion yield in the blue-shifted channel (650–690 nm) reaches a plateau around 5-fold. (c) Zoom in to the first interval, up to 5 mW of 775 nm light, fitted with a power function $y = 1 + mx^{\alpha}$ (yellow solid line). Each data point is the mean, and error bars show SD, of three independent repetitions.

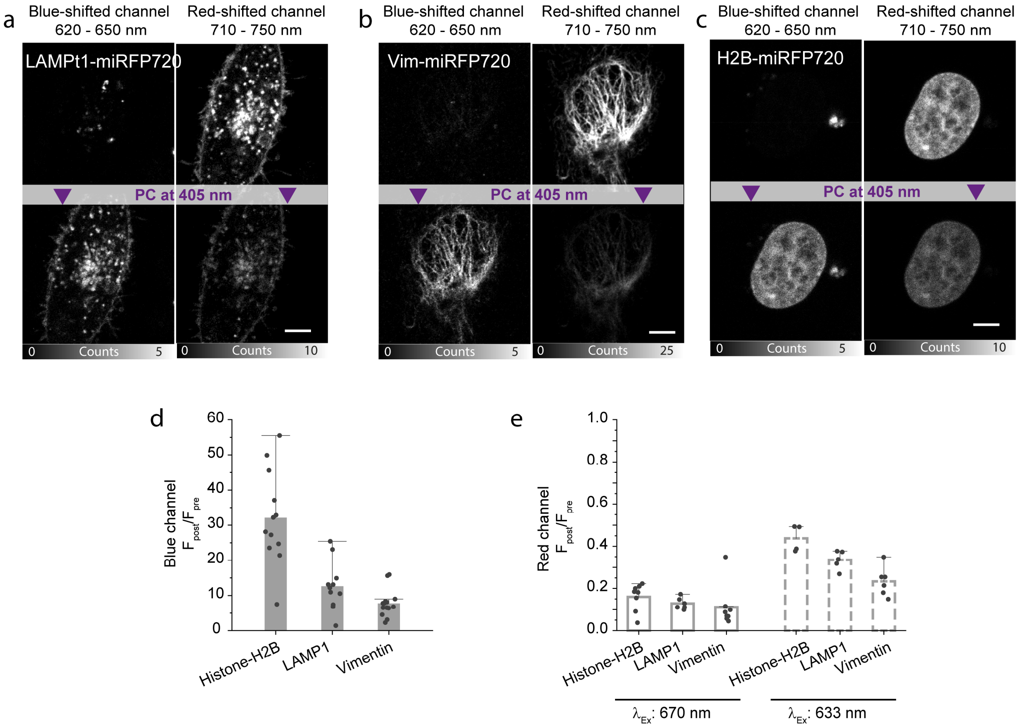

Supplementary Figure 24. Photoconversion in mammalian cells with decoupled excitation for the NIR and far-red form of miRFP720. (a–c) Representative images of photoconversion for miRFP720 tagged to lysosomes (LAMP1-miRFP720) (a), vimentin (vimentin-miRFP720) (b) and histones (H2B-miRFP720) (c). The NIR form is recorded in the interval 710–750 nm and excited at 633 nm (red-shifted channel, right), while the far-red form is recorded at 620–660 nm and excited at 594 nm (blue-shifted channel, left). The recording is sequential for the two forms and the photoconversion is triggered by 405 nm illumination. Scale bar, 5 µm. (d) Photoconversion yield for the blue-shifted channel for the three structures. (e) Change of fluorescence in the red-shifted channel for either 670 nm or 633 nm excitation light. Moving the excitation toward shorter wavelengths increases the crosstalk between the two forms in the red channel. The images are representative of the statistical pool reported in the bar plots, where bars show mean, error bars show SD, for N ≥10 independent repetitions.

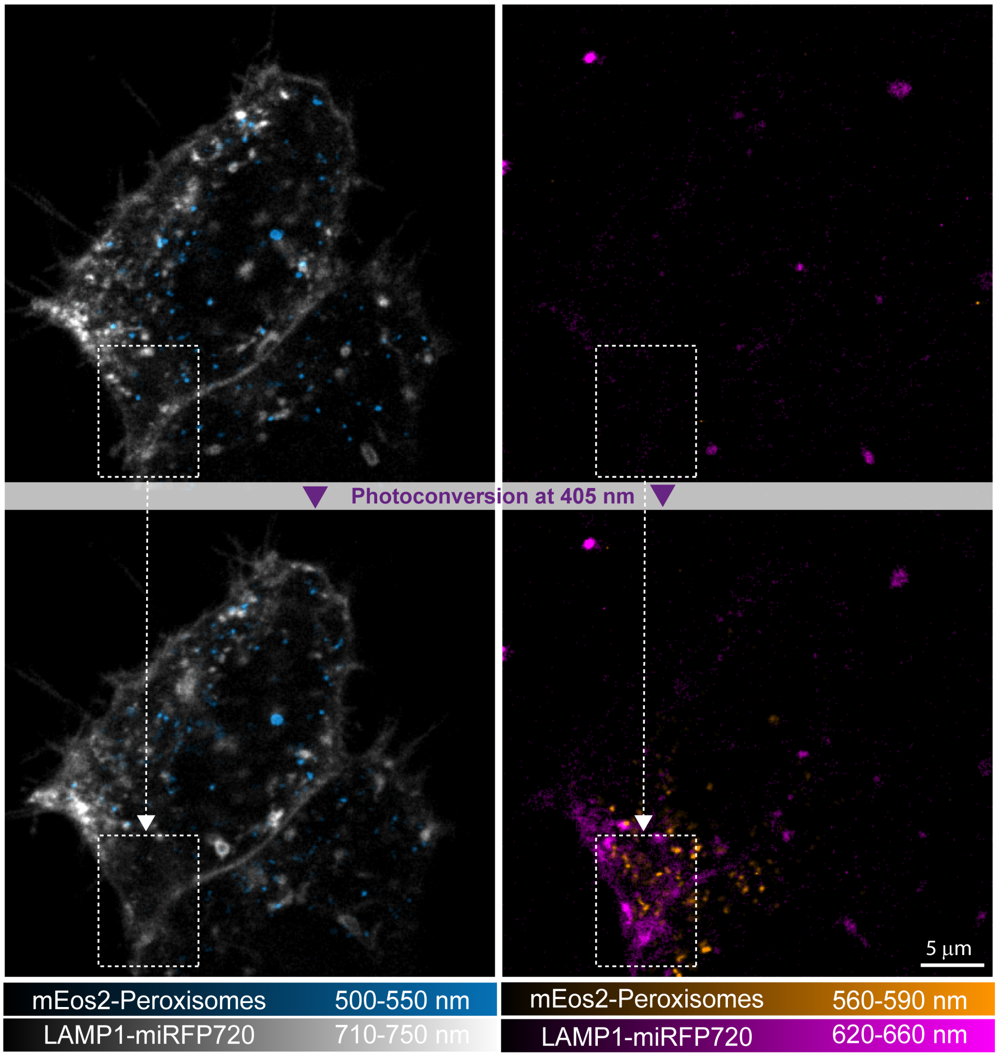

Supplementary Figure 25. Complementary views on the data reported in Fig. 4g–i. Images showing the variation of fluorescence in the four different channels before (top) and after (bottom) photoconversion induced by 405 nm illumination.

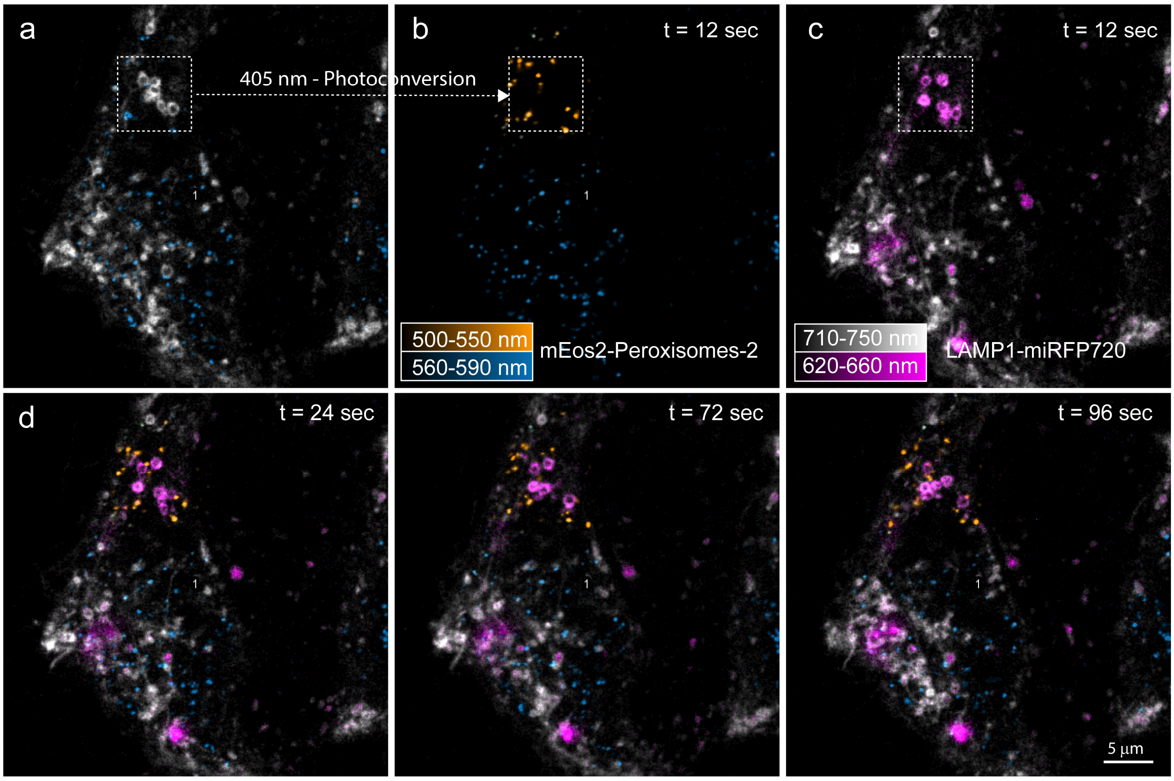

Supplementary Figure 26. Additional examples of multiplexing photoconversion in a spatially common area through 405 nm illumination. (a) Before photoconversion and (b–c) after photoconversion in the marked square, for the ground forms (blue, gray) and photoconverted forms (orange, magenta) of mEos2 (b) and miRFP720 (c), labelling peroxisomes (mEos2-Peroxiomes-2) and lysosomes (LAMP1-miRFP720) respectively. (d) Subsequent timelapse of the four detection channels at a frame rate of 12 s, where all the forms of the PCFPs are visually overlapped.

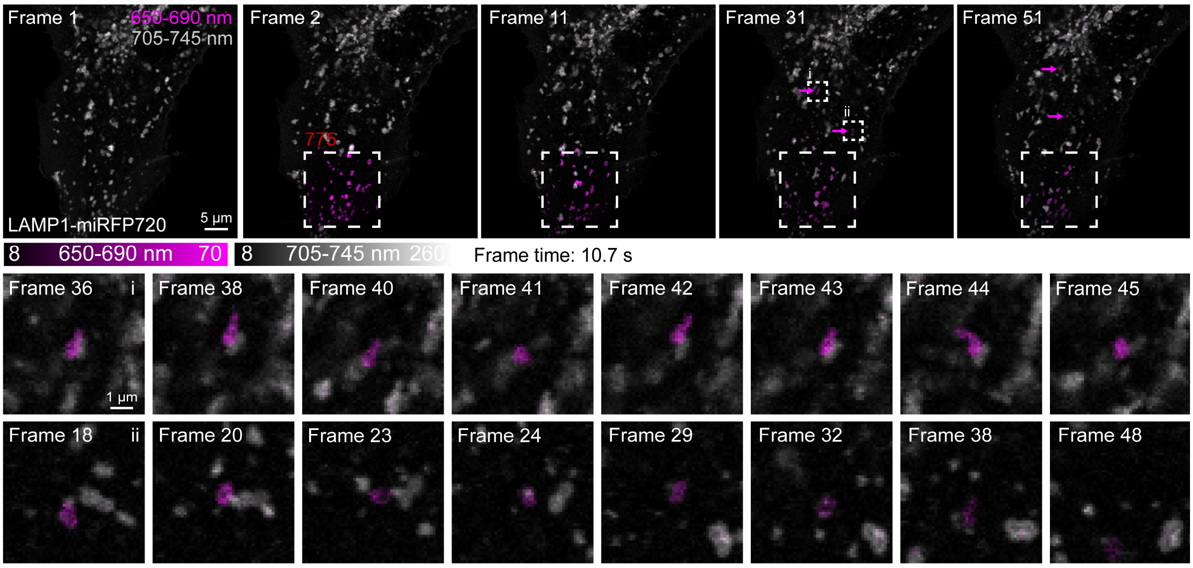

Supplementary Figure 27. Additional zoom-in of the time-lapse shown in Fig. 5, where lysosomes labeled with miRFP720 (LAMP1-miRFP720) in live U2OS cells is reported. The blue-shifted channel (650–690 nm) and red-shifted channel (705–745 nm) are reported as magenta and grey colors respectively. The 20 × 20 μm^2 area enclosed in the white dotted square was photoconverted by illumination with 775 nm light (top raw). This illumination corresponds to a STED image with a cumulative depletion illumination intensity of ~ 1 kJ/cm^2 at each point. To highlight the rearrangement of the vesicles, the dynamics in two smaller roi outside of the photoconverted area is reported (middle and bottom raw). Scale bars, 5 µm and 1 µm.

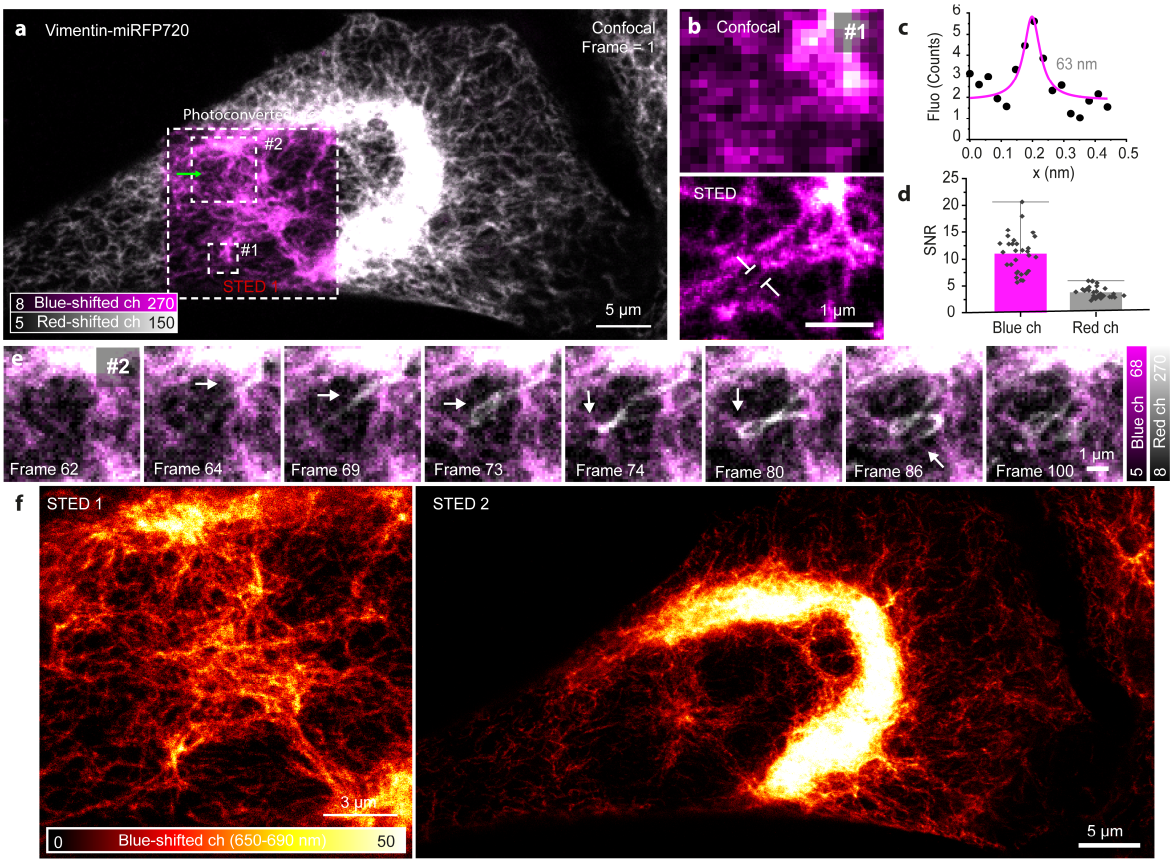

Supplementary Figure 28. Vimentin network dynamics. (a) Vimentin-miRFP720 fusion protein was used to label the vimentin cytoskeleton. Upon photoconversion, driven by the recording of a STED frame in an area of 20 × 20 μm^2, it is possible to follow the invasion into the area of the filaments that were outside of it over time, as for the example (e) in the area marked by box #2 in (a). (b–c) A detail of the network, box #1 in (a), is highlighted to show the improved resolution in STED as compared to confocal imaging. (d) The signal-to-noise (SNR) ratio for the two channels, also reported in the graph of **Fig. 4c**. Data points are from analyzed line profiles across 30 filaments in the images recorded in the blue and red channels. The SNR is calculated by measuring the ratio between the intensity of the center of the filament and the standard deviation of the background around it. (f) STED images of the first (STED 1) and last (STED 2) frame of 100 acquired frames are shown.

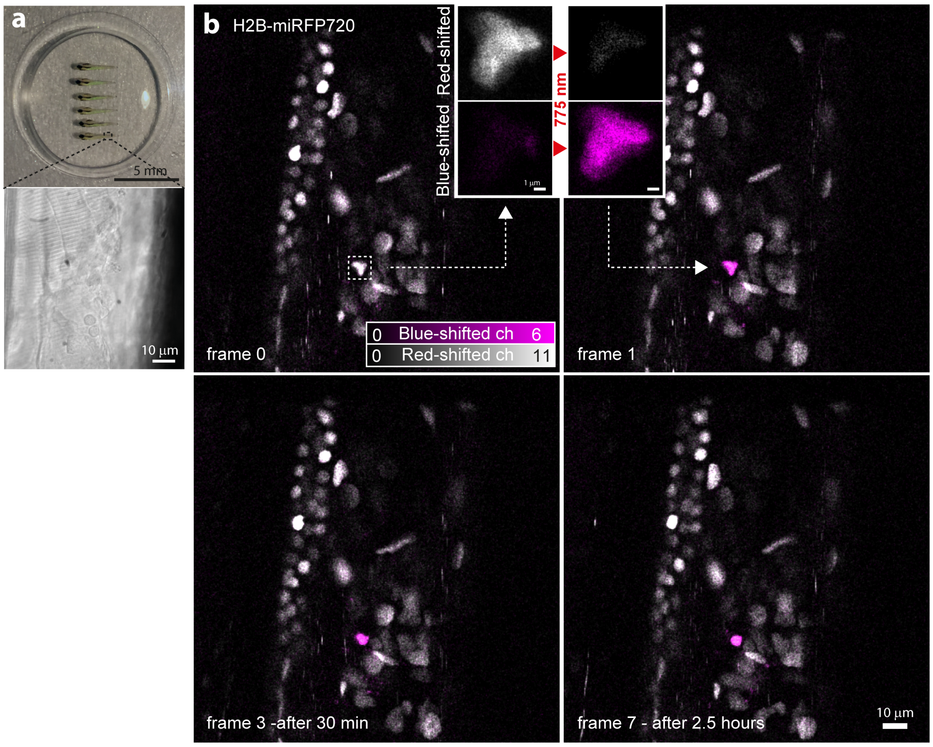

Supplementary Figure 29. Additional frames for the timelapse imaging reported in Fig. 6. (a) In the top image, a representation of the zebrafish and the region used for imaging. In the bottom image, transmission light image for the area of interest, as marked by the box in the top image. (b) Different time points, with a frame interval of 30 min, for the live imaging of the zebrafish, where histones have been labelled through H2B-miRFP720. After the first frame, the nuclei highlighted in the dotted rectangle and zoomed-in in the inset is photoconverted using 775 nm illumination.

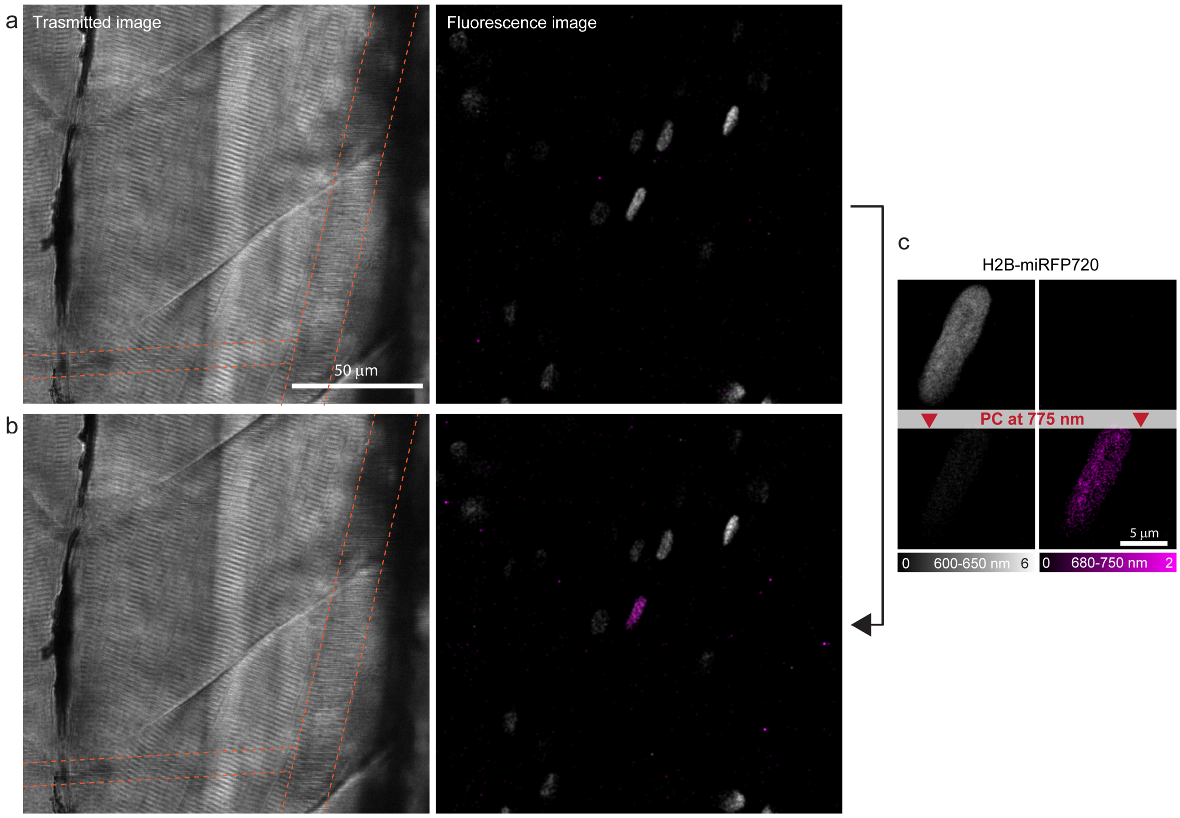

Supplementary Figure 30. Assessment of photodamage for zebrafish larvae imaging. Zebrafish larvae with a mosaic expression of miRFP720-tagged histone (H2B-miRFP720). The area of imaging corresponds to the aorta-gonad-mesonephros region. Before (a) and after (b) photoconversion, the fluorescence (right image) and transmission (left image) images have been acquired to assess the integrity of the tissue. (c) An area of ~ 10 × 10 µm^2 was photoconverted with 775 nm at 7.9. kW/cm^2.

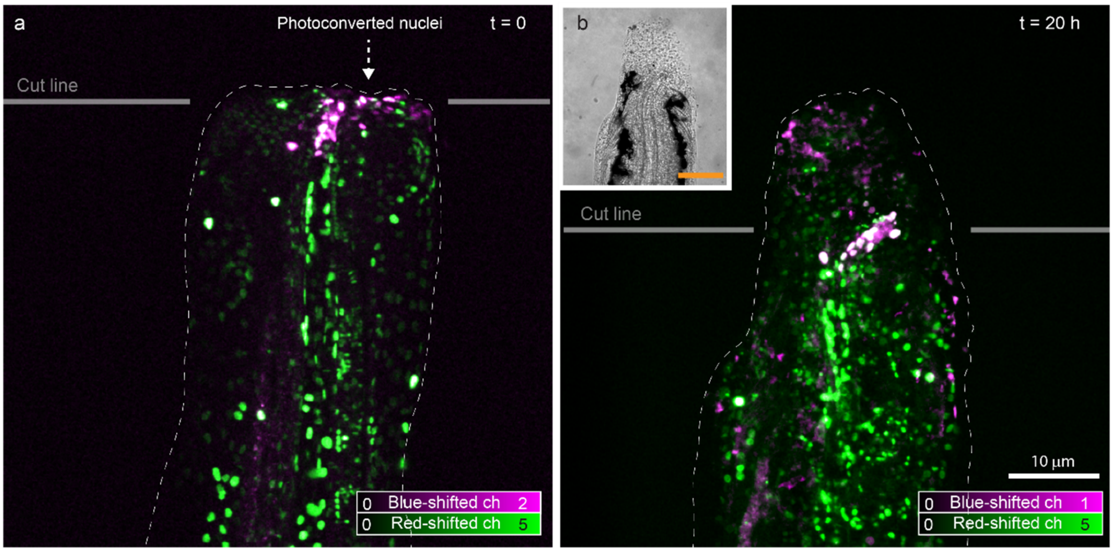

Supplementary Figure 31. Phototoxicity in vivo. Amputate the tail fin of zebrafish larvae at 72 hpf (3dpf, see Morales & Allende, 2019; PMID: 30891030) expressing miRFP720-H2B. The photoconverted channel is in magenta, while the non-photoconverted is in green. The same tail region was imaged immediately after photoconversion (a) and after 20 hours (b). The transmitted image is also presented for the second time point. Around 40 nuclei have been photoconverted in the indicated region by illumination with 775 nm light (40 MHz, 550 ps). For each nucleus, a region of 15 × 15 µm2 have been photoconveterd at 21 MW/cm2 in three consecutive planes at 1 µm axial distance. The presented images are projections of ~50 µm in depth, of images collected at axial distances of 5 µm. Between the photoconversion and imaging 20 h after, the larvae were kept mounted in 0.5 % agarose at 28 °C, which partially affected the normal growth and shape of the regenerating tail fin.

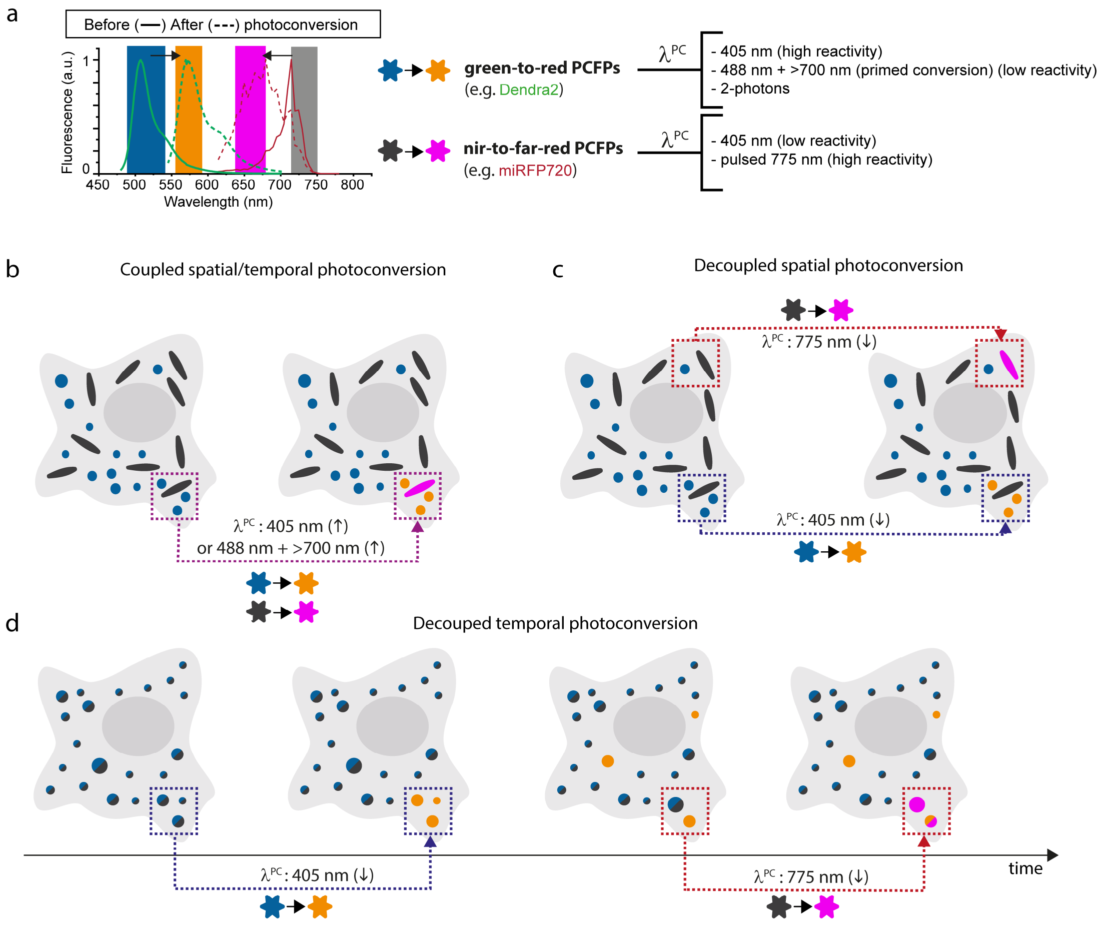

Supplementary Figure 32. Graphical representation of possible photoconversion multiplexing strategies. (a) The combination of green-to-red and NIR-to-far-red PCFPs rely on their spectral separation. Here shown are spectra before (solid lines) and after (dashed lines) photoconversion, of a green-to-red PCFP (green) and an miRFP (red). The four detection channels indicated are detecting the ground forms of the green-to-red PCFP (blue) and miRFP (gray), as well as the photoconverted forms of the green-to-red PCFP (orange) and miRFP (magenta). Additional flexibility is achieved by the possibility to drive the photoconversion with different wavelengths and illumination intensities (right), with combinations unique for the different groups and therefore selective to the specific PCFPs. (b) When PCFPs are located on different subcellular structures, a common photoconversion wavelength and illumination intensity can be used to couple their photoconversion spatiotemporally. (c) Alternatively, selective wavelengths and illumination intensities can act on different PCFPs located in spatially decoupled locations in the cell, simultaneously or sequentially. (d) If the two PCFPs label the same structure of interest, the use of two different and unique photoconversion wavelengths and illumination intensities can report on multiple time points for the dynamic evolution of the protein of interest.

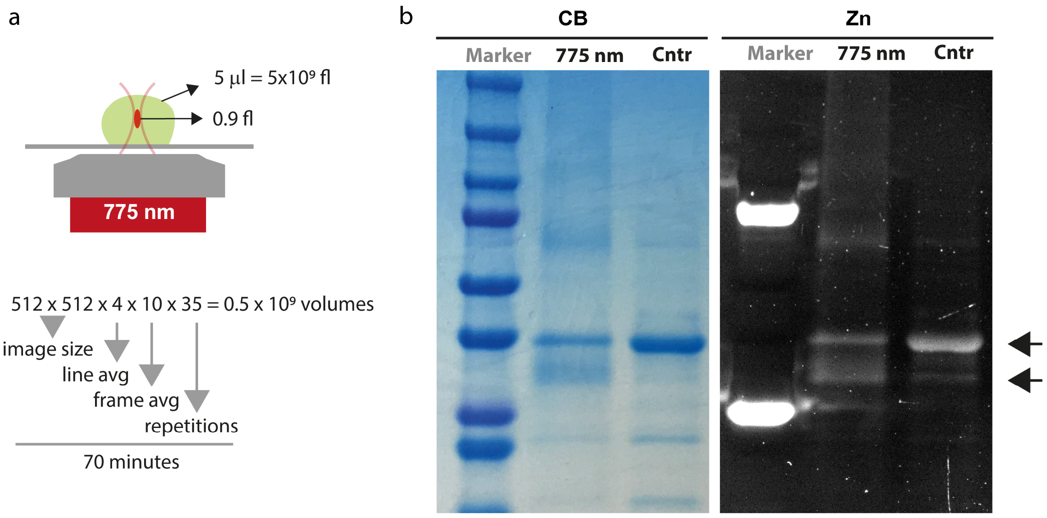

Supplementary Figure 33. Bulk photoconversion of purified miRFP720. (a) Schematic description of the bulk photoconversion of the miRFP720 drop using 775 nm light. In total, 70 min illumination for a total of 0.5×10^9 illumination volumes, allowed a near-complete photoconversion of the drop. (b) Coomassie blue (CB) and $ZnCl_2$ staining for purified miRFP720 in the denatured SDS-PAGE. CB staining visualizes the amount of the protein, while $ZnCl_2$ staining confirms the presence of BV covalently bound to the protein. Arrows indicate the bands of the stained protein before (Cntr) and after (775 nm) photoconversion. One may see the intensity increase of the band having the higher electrophoretic mobility (lower arrow) and the intensity decrease of the band having the lower electrophoretic mobility (upper arrow) after the photoconversion.

Supplementary references

1. Waldchen, S., Lehmann, J., Klein, T., Van De Linde, S. & Sauer, M. Light-induced cell damage in live-cell super-resolution microscopy. *Sci Rep* **5**, (2015).

2. Kilian, N. *et al.* Assessing photodamage in live-cell STED microscopy. *Nature Methods* vol. 15 755–756 Preprint at https://doi.org/10.1038/s41592-018-0145-5 (2018).

3. Alvelid, J. & Testa, I. Fluorescence microscopy at the molecular scale. *Current Opinion in Biomedical Engineering* vol. 12 34–42 Preprint at https://doi.org/10.1016/j.cobme.2019.09.009 (2019).

4. Diaspro, A. *et al.* Multi-photon excitation microscopy. *BioMedical Engineering Online* vol. 5 Preprint at https://doi.org/10.1186/1475-925X-5-36 (2006).

5. Hopt, A. & Neher, E. Highly nonlinear photodamage in two-photon fluorescence microscopy. *Biophys J* **80**, 2029–2036 (2001).

6. Tirlapur, U. K., König, K., Peuckert, C., Krieg, R. & Halbhuber, K. Femtosecond near-infrared laser pulses elicit generation of reactive oxygen species in mammalian cells leading to apoptosis-like death. *Exp Cell Res* **263**, 88–97 (2001).

7. Chudakov, D. M. *et al.* Photoswitchable cyan fluorescent protein for protein tracking. *Nat Biotechnol* **22**, 1435–1439 (2004).

8. Zhang, M. *et al.* Rational design of true monomeric and bright photoactivatable fluorescent proteins. *Nat Methods* **9**, 727–729 (2012).

9. McEvoy, A. L. *et al.* mMaple: A Photoconvertible Fluorescent Protein for Use in Multiple Imaging Modalities. *PLoS One* **7**, (2012).

10. Gurskaya, N. G. *et al.* Engineering of a monomeric green-to-red photoactivatable fluorescent protein induced by blue light. *Nat Biotechnol* **24**, 461–465 (2006).

11. Habuchi, S., Tsutsui, H., Kochaniak, A. B., Miyawaki, A. & van Oijen, A. M. mKikGR, a monomeric photoswitchable fluorescent protein. *PLoS One* **3**, 1–9 (2008).

12. Ando, R. *et al. An optical marker based on the UV-induced green-to-red photoconversion of a fluorescent protein*. vol. 99 www.pnas.orgcgidoi10 (2002).

13. Subach, O. M. *et al.* A photoswitchable orange-to-far-red fluorescent protein, PSmOrange. *Nat Methods* **8**, 771–777 (2011).

14. Lambert, T. J. FPbase: a community-editable fluorescent protein database. *Nat Methods* **16**, 277–278 (2019).
