# Supplementary material for: Blue-shift photoconversion of near-infrared fluorescent proteins for labeling and tracking in living cells and organisms
Source: Nat Commun. 2023 Dec 19;14:8402. doi: 10.1038/s41467-023-44054-9 (PMC10730883; doi:10.1038/s41467-023-44054-9)
Supplement: Supplementary file 3 — Reporting Summary [file 41467_2023_44054_MOESM3_ESM.pdf]

nature portfolio

Corresponding author(s): Ilaria Testa; Francesca Pennacchietti

Last updated by author(s): Oct 4, 2023

Reporting Summary

Nature Portfolio wishes to improve the reproducibility of the work that we publish. This form provides structure for consistency and transparency in reporting. For further information on Nature Portfolio policies, see our Editorial Policies and the Editorial Policy Checklist.

Statistics

For all statistical analyses, confirm that the following items are present in the figure legend, table legend, main text, or Methods section.

n/a	Confirmed	
☐	☒	The exact sample size (*n*) for each experimental group/condition, given as a discrete number and unit of measurement
☐	☒	A statement on whether measurements were taken from distinct samples or whether the same sample was measured repeatedly
☐	☒	The statistical test(s) used AND whether they are one- or two-sided<br>*Only common tests should be described solely by name; describe more complex techniques in the Methods section.*
☒	☐	A description of all covariates tested
☒	☐	A description of any assumptions or corrections, such as tests of normality and adjustment for multiple comparisons
☐	☒	A full description of the statistical parameters including central tendency (e.g. means) or other basic estimates (e.g. regression coefficient) AND variation (e.g. standard deviation) or associated estimates of uncertainty (e.g. confidence intervals)
☐	☒	For null hypothesis testing, the test statistic (e.g. *F*, *t*, *r*) with confidence intervals, effect sizes, degrees of freedom and *P* value noted<br>*Give P values as exact values whenever suitable.*
☒	☐	For Bayesian analysis, information on the choice of priors and Markov chain Monte Carlo settings
☒	☐	For hierarchical and complex designs, identification of the appropriate level for tests and full reporting of outcomes
☒	☐	Estimates of effect sizes (e.g. Cohen's *d*, Pearson's *r*), indicating how they were calculated

Our web collection on statistics for biologists contains articles on many of the points above.

Software and code

Policy information about availability of computer code

Data collection	The imaging was performed in several microscopes: (1) open-source hardware and acquisition control software ImSwitch in Python and Imspector v0.10 (2) Leica SPS microscope, LASX software, Zeiss Confocal software
Data analysis	Data analysis was performed with custom-written scripts in Fiji (ImageJ v1.53c), Origin Pro 2021 and 2022, Matlab R2019b. StarDist Colab notebook in ZeroCostDL4Mic has been used for bacteria segmentation, the training dataset is provided. The scripts are available in the Zenodo repository "Dataset for "Blue-shift photoconversion of near-infrared fluorescent proteins for labeling and tracking in living cells and organisms" at doi.org/10.5281/zenodo.5884554.

For manuscripts utilizing custom algorithms or software that are central to the research but not yet described in published literature, software must be made available to editors and reviewers. We strongly encourage code deposition in a community repository (e.g. GitHub). See the Nature Portfolio guidelines for submitting code & software for further information.

Data

Policy information about availability of data

All manuscripts must include a data availability statement. This statement should provide the following information, where applicable:

- Accession codes, unique identifiers, or web links for publicly available datasets
- A description of any restrictions on data availability
- For clinical datasets or third party data, please ensure that the statement adheres to our policy

All data supporting the findings of this study are available in the Zenodo repository "Dataset for "Blue-shift photoconversion of near-infrared fluorescent proteins for labeling and tracking in living cells and organisms" at doi.org/10.5281/zenodo.5884554.

Research involving human participants, their data, or biological material

Policy information about studies with human participants or human data. See also policy information about sex, gender (identity/presentation), and sexual orientation and race, ethnicity and racism.

Reporting on sex and gender	Non applicable to this study
Reporting on race, ethnicity, or other socially relevant groupings	Non applicable to this study
Population characteristics	Non applicable to this study
Recruitment	Non applicable to this study
Ethics oversight	Non applicable to this study

Note that full information on the approval of the study protocol must also be provided in the manuscript.

Field-specific reporting

Please select the one below that is the best fit for your research. If you are not sure, read the appropriate sections before making your selection.

☒ Life sciences ☐ Behavioural & social sciences ☐ Ecological, evolutionary & environmental sciences

For a reference copy of the document with all sections, see nature.com/documents/nr-reporting-summary-flat.pdf

Life sciences study design

All studies must disclose on these points even when the disclosure is negative.

Sample size	No sample size dependent statistical testing was performed.<br>The study report on a photoconversion mechanism and its applicability in live cell imaging. The sample size has been chosen in order to account for differences and variability in the illumination conditions. To assess the invariability of the photoconversion mechanism to the biological system in which the protein is expressed, the same illumination dependency measurement has been performed.
Data exclusions	No data where excluded from the analysis
Replication	The phenomena of photoconversion has been demonstrated in three different systems: bacteria (E. coli), mammalian cells (Hela cell and U2OS cells) and organism (Zebrafish embryo). Its wavelength, power and time dependency were consistent in different samples and day of measurements with N≥3.
Randomization	No allocation into experimental group were performed
Blinding	No allocation into experimental group were performed

Reporting for specific materials, systems and methods

We require information from authors about some types of materials, experimental systems and methods used in many studies. Here, indicate whether each material, system or method listed is relevant to your study. If you are not sure if a list item applies to your research, read the appropriate section before selecting a response.

Materials & experimental systems

n/a	Involved in the study	
☒	☐	Antibodies
☐	☒	Eukaryotic cell lines
☒	☐	Palaeontology and archaeology
☐	☒	Animals and other organisms
☒	☐	Clinical data
☒	☐	Dual use research of concern
☒	☐	Plants

Methods

n/a	Involved in the study	
☒	☐	ChIP-seq
☒	☐	Flow cytometry
☒	☐	MRI-based neuroimaging

Eukaryotic cell lines

Policy information about cell lines and Sex and Gender in Research

Cell line source(s)	U2OS (ATCC HTB-96) and Hela cells (ATCC CCL-2)
Authentication	No authentication
Mycoplasma contamination	Not tested
Commonly misidentified lines (See ICLAC register)	No commonly misidentified cell lines were used in the study

Animals and other research organisms

Policy information about studies involving animals; ARRIVE guidelines recommended for reporting animal research, and Sex and Gender in Research

Laboratory animals	Zebrafish (Danio rerio, AB strain), 4 dpf
Wild animals	No wild animals were used.
Reporting on sex	Not applicable to this study
Field-collected samples	No field collected samples were used.
Ethics oversight	Zebrafish (Danio rerio, AB strain) were kept in the Karolinska Institutet Zebrafish core facility according to standard protocols and under the ethical permits Nr 5756/17 and 14049/19, approved by the Stockholm Ethical Committee (Stockholms djurförsöksetiska nämnd) and issued by the Swedish Board of Agriculture.

Note that full information on the approval of the study protocol must also be provided in the manuscript.
